# Actin remodeling mediates ROS production and JNK activation to drive apoptosis-induced proliferation

**DOI:** 10.1371/journal.pgen.1010533

**Published:** 2022-12-05

**Authors:** Luchi Farrell, Aleix Puig-Barbe, Md. Iqramul Haque, Alla Amcheslavsky, Mengyuan Yu, Andreas Bergmann, Yun Fan

**Affiliations:** 1 University of Birmingham, School of Biosciences, Birmingham, United Kingdom; 2 Department of Physiology, Faculty of Veterinary Science, Bangladesh Agricultural University, Mymensingh, Bangladesh; 3 University of Massachusetts Medical School, Department of Molecular, Cell and Cancer Biology, Worcester, Massachusetts, United States of America; University of Michigan, UNITED STATES

## Abstract

Stress-induced cell death, mainly apoptosis, and its subsequent tissue repair is interlinked although our knowledge of this connection is still very limited. An intriguing finding is apoptosis-induced proliferation (AiP), an evolutionary conserved mechanism employed by apoptotic cells to trigger compensatory proliferation of their neighboring cells. Studies using *Drosophila* as a model organism have revealed that apoptotic caspases and c-Jun N-terminal kinase (JNK) signaling play critical roles to activate AiP. For example, the initiator caspase Dronc, the caspase-9 ortholog in *Drosophila*, promotes activation of JNK leading to release of mitogenic signals and AiP. Recent studies further revealed that Dronc relocates to the cell cortex via Myo1D, an unconventional myosin, and stimulates production of reactive oxygen species (ROS) to trigger AiP. During this process, ROS can attract hemocytes, the *Drosophila* macrophages, which further amplify JNK signaling cell non-autonomously. However, the intrinsic components connecting Dronc, ROS and JNK within the stressed signal-producing cells remain elusive. Here, we identified LIM domain kinase 1 (LIMK1), a kinase promoting cellular F-actin polymerization, as a novel regulator of AiP. F-actin accumulates in a Dronc-dependent manner in response to apoptotic stress. Suppression of F-actin polymerization in stressed cells by knocking down LIMK1 or expressing Cofilin, an inhibitor of F-actin elongation, blocks ROS production and JNK activation, hence AiP. Furthermore, Dronc and LIMK1 genetically interact. Co-expression of Dronc and LIMK1 drives F-actin accumulation, ROS production and JNK activation. Interestingly, these synergistic effects between Dronc and LIMK1 depend on Myo1D. Therefore, F-actin remodeling plays an important role mediating caspase-driven ROS production and JNK activation in the process of AiP.

## Author summary

In multicellular organisms, damaged cells are frequently removed via apoptosis, the major form of programmed cell death. Intriguingly, these apoptotic cells can emit signals to induce proliferation of their neighboring cells for the maintenance of tissue homeostasis, a phenomenon termed apoptosis-induced proliferation (AiP). Caspases, a family of cysteine proteases well known to execute apoptosis, also play a critical role to trigger AiP via activation of JNK, a stress response kinase. In this study, we identified the actin cytoskeleton, a dynamic structural network of the cell, as a key mediator of JNK activation in AiP. During this process, actin filaments undergo increased polymerization which depends on LIM domain kinase 1 (LIMK1). Genetically, caspases and LIMK1 work together to promote actin polymerization, which in turn drives production of reactive oxygen species (ROS) and the subsequent activation of JNK. Therefore, our study discovered a role of actin remodeling in dying cells which mediates the non-apoptotic roles of caspases in AiP and tissue repair.

## Introduction

In multicellular organisms, damaged cells are frequently removed by apoptosis, the major form of programmed cell death. Intriguingly, these stress-induced dying cells, prior to their removal, can actively induce proliferation of neighboring cells to compensate for their loss and maintain tissue integrity [1–8]. This phenomenon has been termed as apoptosis-induced compensatory proliferation or apoptosis-induced proliferation (AiP) [9, 10]. Importantly, AiP is found not only to promote tissue recovery and regeneration, but also to drive tumor development and cancer recurrence [4, 11–13]. Therefore, understanding the mechanisms of AiP has significant clinical and therapeutic implications.

Caspases, an evolutionary conserved family of cysteine proteases, are essential for both apoptosis and AiP. Whilst caspases mediate execution of apoptosis, they can also promote AiP via activation of mitogenic signals such as cytokines or growth signaling pathways in a context-dependent manner [1, 4, 5, 14–16]. Studies in *Drosophila* have revealed distinct mechanisms of AiP mediated by different groups of caspases. In differentiating tissues where cells have exited mitosis, the caspase-3-like effector caspases DrICE and Dcp-1 activate Hedgehog signaling to drive cell cycle re-entry [14]. In contrast, in proliferating tissues where cells are actively dividing, the caspase-9-like initiator caspase Dronc activates the c-Jun N-terminal kinase (JNK), an evolutionary conserved stress response molecule, which in turn induces growth signals such as Wg, Dpp and EGFR signaling resulting in AiP [1, 15, 17].

Notably, in the proliferating larval eye and wing epithelia, Reactive Oxygen Species (ROS), the oxygen-containing free radicals produced during cellular metabolism, accumulate in apoptotic tissues and trigger activation of JNK [18, 19]. Recent studies further showed that ROS and JNK in apoptotic cells also damage the epithelial basement membrane and act as signals to recruit macrophage-like hemocytes, which in turn contribute to further activation of JNK signaling cell non-autonomously [20–22]. Interestingly, the initiator caspase Dronc translocates from cytosol to the plasma membrane, where it exerts its non-apoptotic function to activate Duox, a NADPH oxidase, for ROS production [20]. Myo1D, an unconventional myosin, is critical for this process through its interaction with Dronc. However, it remains unknown what mediates this non-apoptotic action of Dronc and Myo1D to drive ROS production and JNK activation within the dying cells.

**Fig 1:**
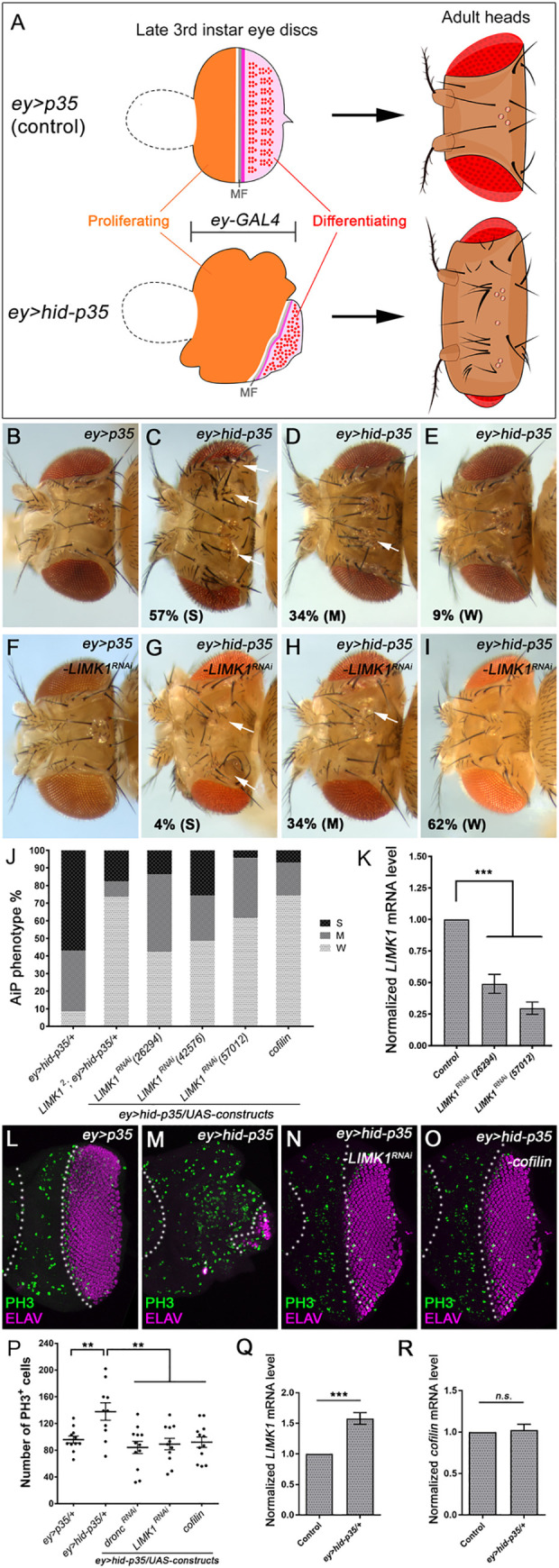
LIMK1 and Cofilin, regulators of F-actin polymerization, are required for AiP. (A) A schematic representation of the AiP-dependent overgrowth assay. *ey-GAL4* is expressed in the developing larval eye disc which is composed of the anterior proliferating tissue and the posterior differentiating tissue, separated by a morphogenic furrow (MF, the grey bar). Compared to the control *ey>p35* (*ey-GAL4 UAS-p35*), simultaneously expression of *hid* and *p35* in *ey>hid-p35* (*ey-GAL4 UAS-hid UAS-p35*) results in AiP-dependent overgrowth of the anterior proliferating tissue, which leads to an adult head overgrowth phenotype characterized by expanded head cuticle with ectopic sensory organs such as ocelli and bristles. Consequently, sizes of the differentiating eye tissue and adult eye are reduced in *ey>hid-p35* animals. These larval and adult phenotypes were used as readouts of AiP in our analyses. (B-I) Representative adult fly head images of the indicated genotypes. Compared to the control *ey>p35* (B), which is similar to wildtype, *ey>hid-p35* fly head capsules display overgrowth phenotypes which can be grouped into three categories (C-E): severe (S), moderate (M) and weak (W, including wildtype-like), as previously described [15]. A majority of *ey>hid-p35* flies show either severe (57%) or moderate (34%) overgrowth phenotype, characterized by overgrown head capsules with duplications of sensory organs including bristles and ocelli (C and D, arrows). (F) Knockdown of *LIMK1* by RNAi does not cause any defects. (G-I) *LIMK1*^*RNAi-57012*^ strongly reduces the percentage of *ey>hid-p35* flies displaying severe (4%) and moderate overgrowth (34%) phenotypes, with a large increase of flies (62%) showing a weak phenotype or wildtype-like appearance. (J) Summary of the suppression of the *ey>hid-p35* overgrowth phenotype by a *LIMK1* hypomorphic mutant (*LIMK1*^*2*^), expressing three independent *LIMK1*^*RNAi*^ lines (26294, 42576 and 57012) or *cofilin*. Black indicates severe, dark grey indicates moderate, and light grey indicates weak or wildtype-like phenotypes. (K) Specificity and efficiency of LIMK1 RNAi lines were determined by measuring *LIMK1* transcript levels with RT-qPCR. Compared to the control without expression of *LIMK1*^*RNAi*^, two independent *LIMK1*^*RNAi*^ lines, 26294 and 57012, suppress *LIMK1* transcript levels to less than 50% and 30%, respectively. These reductions are statistically significant (***P<0.001). (L-O) Late 3^rd^ instar eye discs labelled with the mitotic marker PH3 (green) and the photoreceptor neuron marker ELAV (magenta), anterior is to the left. ELAV is used to mark the posterior differentiating portion of the eye discs. White dotted lines indicate the anterior proliferating portion of the eye discs. Compared to the control *ey>p35* (L), the size of the anterior proliferating portion and the number of mitotic cells increase in the *ey>hid-p35* eye discs (M). These increases are suppressed by expressing *LIMK1*^*RNAi-57012*^ (N) or *cofilin* (O). (P) Quantification of the number of PH3^+^ cells in the anterior portion of the eye discs of the indicated genotypes. Compared to *ey>p35*, the number of PH3^+^ cells are significantly (**p < 0.01) increased in the *ey>hid-p35* discs. This increase is significantly (**p < 0.01) reduced in response to expression of *dronc*^*RNAi*^, *LIMK1*^*RNAi*^ or *cofilin*. (Q and R) Expression levels of *LIMK1* (Q) and *cofilin* (R) were measured by RT-qPCR. Compared to the control *ey>p35-mCherry* (*ey-GAL4 UAS-p35 UAS-mCherry*) eye discs, expression of *LIMK1* is increased at about 1.6-fold in *ey>hid-p35* (***P<0.001). In contrast, expression of *cofilin* is not significantly changed.

**Fig 2:**
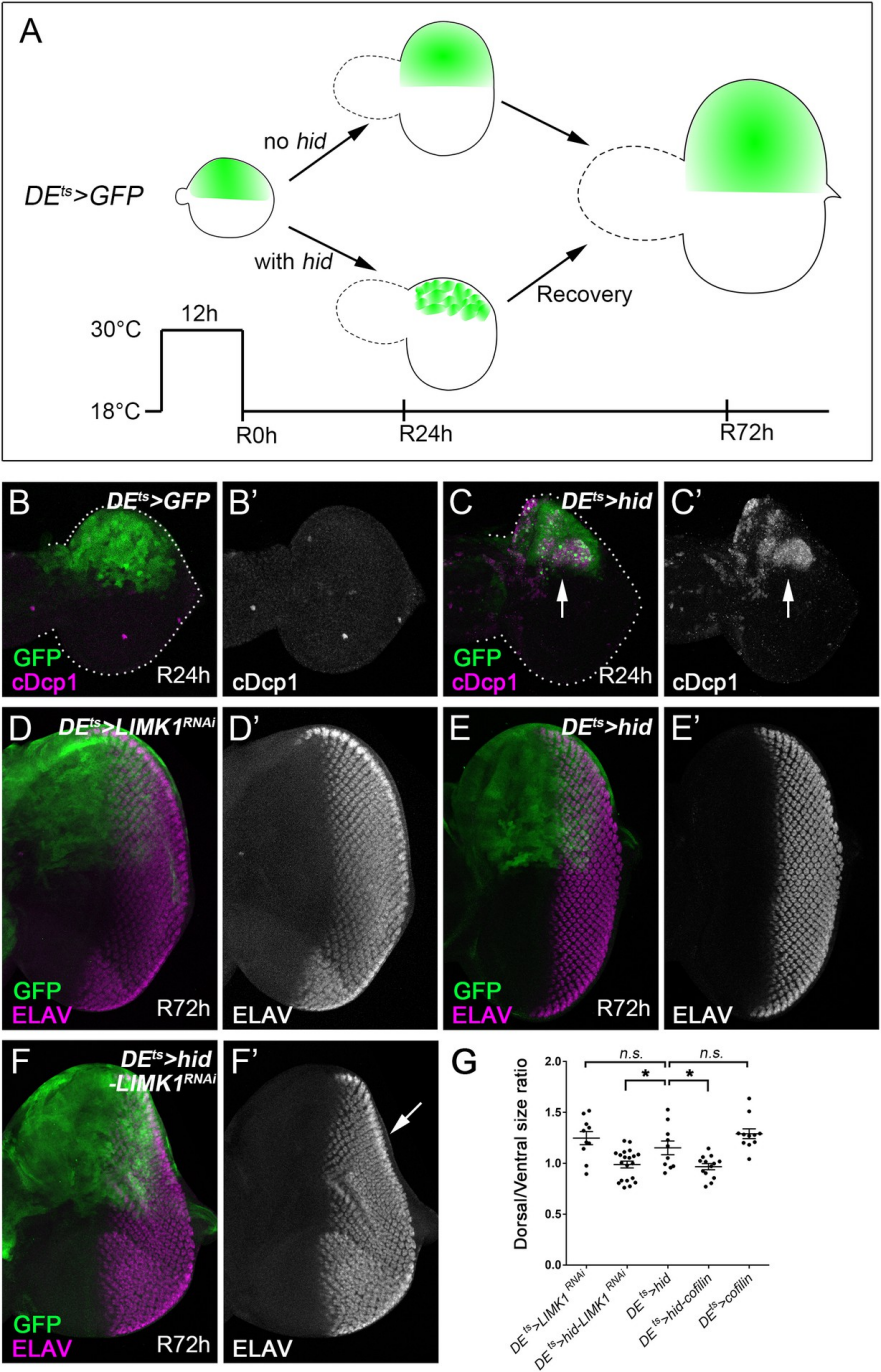
LIMK1 is required for complete tissue regeneration in response to apoptosis. (A) A schematic representation of the AiP-dependent regeneration assay. Conditional expression of GFP and the pro-apoptotic gene *hid* is under control of the *Dorsal Eye-GAL4* (*DE-GAL4*) driver, which is expressed in the dorsal half of the eye disc, together with *tub-GAL80*^*ts*^, a ubiquitously expressed and temperature sensitive (ts) inhibitor of GAL4. *tub-GAL80*^*ts*^ inhibits *DE-GAL4* (*DE*^*ts*^) at 18°C, but not at 30°C. In this regeneration assay, a temperature shift to 30°C for 12 hours during second instar larval stage induces expression of GFP together with or without *hid*. This is then followed by a recovery period at 18°C. Compared to the control with GFP expression only, a 12-hour expression of *hid* results in tissue ablation in the dorsal eye half, which is regenerated completely after a recovery period of 72 hours (R72h). (B-C’) Early 3^rd^ instar eye discs, anterior is to the left, were labelled with GFP (green in B, C) and an apoptosis marker cDcp1 (magenta in B, C and grey in B’, C’). Dashed lines highlight the discs. Compared to the control (*DE*^*ts*^*>GFP*, B, B’), conditional expression of *hid* (*DE*^*ts*^*>hid*, C, C’) for 12 hours under the control of *DE*^*ts*^ results in a strong induction of apoptosis (arrows) and loss of bilateral symmetry of the disc which are visible after a recovery period of 24 hours (R24h). (D-F’) Late 3^rd^ instar eye discs, anterior is to the left. ELAV (magenta in D, E, F and grey in D’, E’, F’) labels photoreceptor neurons and is used to outline the shape of the discs. Conditional expression of *LIMK1*^*RNAi*^ (D, D’), *hid* (E, E’) or *hid* and *LIMK1*^*RNAi*^ (F, F’) was under control of *DE*^*ts*^ and indicated by GFP (green in D, E, F). A temperature shift to 30°C for 12 hours was followed by a recovery period of 72 hours at 18°C (R72h). (D, D’) Following this protocol, expression of *LIMK1*^*RNAi*^ alone (*DE*^*ts*^*>LIMK1*^*RNAi*^) does not affect the eye disc morphology indicated by the normal ELAV pattern in the dorsal half of the eye disc. (E, E’) Tissue damage induced by expression of *hid* (*DE*^*ts*^*>hid*) for 12 hours has fully recovered after 72 hours recovery (R72h) at 18°C as indicated by the largely normal ELAV pattern in the late third instar eye discs. (F, F’) A *DE*^*ts*^*>hid* eye disc that simultaneously expresses *LIMK1*^*RNAi*^ (*DE*^*ts*^*>hid-LIMK1*^*RNAi*^). The arrow in (F’) highlights the incomplete ELAV pattern on the dorsal half of the disc indicating that the regenerative response was partially impaired by reduction of LIMK1. (D) Quantification of the dorsal/ventral size ratio in representative eye discs of the indicated genotypes. Compared to *DE*^*ts*^*>hid*, expression of *LIMK1*^*RNAi*^ or *cofilin* significantly (*P < 0.5) reduces the size of the dorsal half of the eye disc. As the controls, disc sizes of *DE*^*ts*^*>LIMK1*^*RNAi*^ and *DE*^*ts*^*>cofilin* are not significantly (n.s.) different from those of *DE*^*ts*^*>hid*.

To study the mechanisms of AiP in proliferating tissues, we have previously developed two genetic assays, an overgrowth assay and a regeneration assay, to identify regulators of AiP [15]. In the overgrowth assay, *hid*, a pro-apoptotic gene, and *p35*, an inhibitor of effector caspases DrICE and Dcp-1, are simultaneously expressed under control of the *ey-GAL4* driver (*ey>hid-p35*, Fig 1A) in the developing larval eye epithelium which is composed of the anterior proliferating and the posterior differentiating tissues. While the posterior differentiating tissue develops to the adult eye, the anterior proliferating tissue develops to adult head appendages including cuticle and sensory organs such as ocelli and bristles. Expression of P35 inhibits the effector caspases DrICE and Dcp-1, therefore AiP in the differentiating tissue, but not the initiator caspase Dronc [14, 23]. Hence, Dronc-dependent AiP occurs specifically in the proliferating eye tissues in *ey>hid-p35*. Cells in this tissue are kept ‘undead’ because apoptotic responses are activated but execution of cell death is blocked. These ‘undead’ cells continuously drive AiP leading to a head overgrowth phenotype, a convenient readout of AiP for genetic screens (Fig 1A). In the regeneration assay, *hid* is expressed in a temporally and spatially controlled manner to induce a pulse of apoptosis therefore tissue ablation. This tissue is then allowed to recover to observe AiP and study its regulation during tissue regeneration (Fig 2A).

The actin cytoskeleton, a structural network found in all eukaryotic cells, consists of actin and actin-binding proteins [24, 25]. Actin exists in two forms: globular monomers (G-actin) and filamentous polymers (F-actin). Actin filaments are highly dynamic and constantly undergo assembly and disassembly. The spatial and temporal regulation of F-actin mediates multiple cellular signaling responses including cell division, motility and phagocytosis. The evolutionary conserved LIM domain kinase 1 (LIMK1) and Cofilin are essential for the formation of actin filaments [26, 27]. Cofilin, also called actin-depolymerizing factor (ADF), promotes rapid F-actin turnover by severing actin filaments [28]. LIMK1 phosphorylates Cofilin and inhibits its activity, therefore promotes F-actin polymerization. Although AiP relies on cell-cell interactions and signal transduction, it is not yet clear whether and how the F-actin network is involved in this process.

Using both overgrowth and regeneration assays, in this study, we showed that knockdown of LIMK1 or overexpression of Cofilin suppresses both AiP-dependent tissue overgrowth and regeneration. Consistent with this, F-actin polymerization, regulated by LIMK1 and Cofilin, was observed in AiP. It depends on the initiator caspase Dronc and is required for activation of JNK and its upstream ROS production. Furthermore, Dronc and LIMK1 genetically interact and drive F-actin remodeling in AiP via Myo1D.

## Results

### LIMK1, a key factor promoting F-actin polymerization, is required for AiP

We have recently shown that Myo1D, an unconventional actin-based motor protein, is critical for AiP [20]. This suggests a potential role of actin dynamics and actin-based motility in AiP. To address this, we examined roles of LIMK1 and Cofilin, two key factors regulating F-actin polymerization, using the *ey>hid-p35* assay (Fig 1A). AiP-dependent overgrowth phenotypes observed in *ey>hid-p35* animals vary and can be categorized into three groups: severe, moderate and weak (Fig 1B–1E, quantified in Fig 1J) [15]. Using this approach, we identified three independent *LIMK1* RNAi lines as suppressors of AiP (Fig 1F–1I, quantified in Fig 1J). Compared to the control *ey>hid-p35* flies, in which 91% show either severe or moderate overgrowth phenotypes, the *LIMK1* RNAi line 57012 suppresses it to 38%. The other two *LIMK1* RNAi lines, 42576 and 26294, suppress it to a lesser degree, with the percentage of severe or moderate overgrowth flies reduced to 51% and 58%, respectively. qPCR measurements suggest that this difference correlates with the strength of the RNAi (Fig 1K). The line 57012 was used in the follow-up experiments because it is the strongest *LIMK1*^*RNAi*^ line. To further confirm that loss-of-LIMK1 suppresses AiP, *LIMK1*^*2*^, a hypomorphic but viable mutant of LIMK1 [29], was used. The *ey>hid-p35*-induced overgrowth phenotype is strongly suppressed in the hemizygous *LIMK1*^*2*^ mutants with only 26% of flies showing the overgrowth phenotype (Fig 1J). As LIMK1 phosphorylates and inhibits Cofilin, we examined the roles of Cofilin for regulation of AiP. Similar to loss of LIMK1, overexpression of Cofilin also strongly suppresses the *ey>hid-p35*-induced overgrowth phenotype (Fig 1J).

To further confirm that the suppression of the *ey>hid-p35* overgrowth phenotype by LIMK1 was due to a reduction of cell proliferation, we examined late 3^rd^ instar larval eye imaginal discs. Compared to the *ey>p35* control, the number of mitotic cells indicated by the PH3 antibodies increases in *ey>hid-p35* discs (Fig 1L and 1M, quantified in Fig 1P). This increase is suppressed by RNAi knockdown of LIMK1 or overexpression of Cofilin (Fig 1N, 1O and 1P). Notably, this suppression is not due to potential roles of LIMK1 and Cofilin in regulation of apoptosis as knockdown of LIMK1 or overexpression of Cofilin does not suppress *hid*-induced apoptosis (S1 Fig). Therefore, LIMK1 and Cofilin, two key regulators of F-actin dynamics, play critical and antagonistic roles in regulating AiP. Furthermore, we measured expression of LIMK1 and Cofilin using RT-qPCR. Interestingly, LIMK1, but not Cofilin, is transcriptionally increased in *ey>hid-p35* discs compared to the control (Fig 1Q and 1R). Altogether, these data suggest that LIMK1 is induced and required for AiP-dependent tissue overgrowth.

Next, we examined whether LIMK1 is required for AiP-dependent regenerative growth by using the regeneration assay (Fig 2A). In this assay, to induce spatially and temporally controlled apoptosis and tissue ablation, the pro-apoptotic gene *hid* was expressed in the dorsal portion of the larval eye disc for 12 hours. This was under the control of the driver *Dorsal Eye-GAL4* (*DE-GAL4*) together with the *tub-GAL80*^*ts*^, a ubiquitously expressed and temperature sensitive (ts) inhibitor of GAL4 [30]. *tub-GAL80*^*ts*^ inhibits activity of *DE-GAL4* at 18°C. A temperature shift to 30°C releases this inhibition and allows expression of *hid*, i.e. induction of apoptosis, in the dorsal half of the eye disc under the control of *DE-GAL4*. After a 12-hour period of *hid* expression, larvae were transferred back to 18°C for tissue recovery. Compared to the control where only GFP is expressed (Fig 2B and 2B’), a clear tissue ablation was observed at 24 hours of recovery (R24h) (Fig 2C and 2C’). This apoptosis-induced tissue ablation can fully regenerate via AiP after 72 hours of recovery (Fig 2E) [15]. However, knockdown of LIMK1 or overexpression of Cofilin hinders such tissue regeneration (Fig 2F, quantified in Fig 2G). As a control, knockdown of LIMK1 or overexpression of Cofilin does not affect the development of larval discs (Fig 2D and 2G). Taken together, LIMK1 and Cofilin were identified as regulators of AiP in both overgrowth and regeneration assays.

### F-actin accumulates in the process of AiP

**Fig 3:**
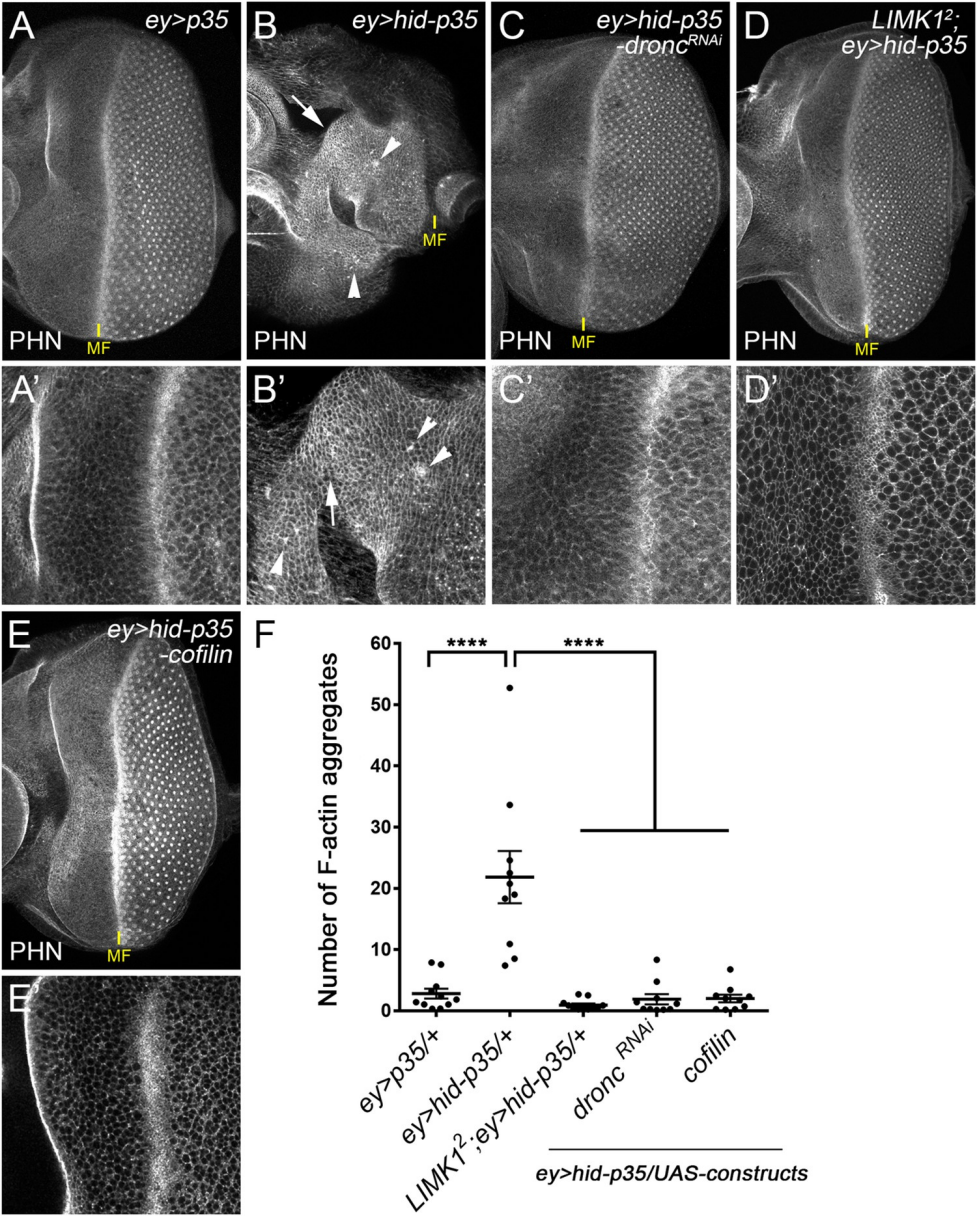
F-actin aggregates increase in the *ey>hid-p35* discs and depends on LIMK1 and Cofilin. (A-E’) Late 3^rd^ instar eye discs labelled with phalloidin (PHN), a marker of F-actin. A’, B’ and C’ are enlarged images of A, B and C, respectively. Compared to the control *ey>p35* (A, A’), bright F-actin signals (arrows) with an increased number of F-actin aggregates (arrowheads) were observed in the overgrown portion of the *ey>hid-p35* discs anterior to the MF (B, B’). These aggregates disappear in response to knockdown of *dronc* with RNAi (C, C’), in *LIMK1*^*2*^ hemizygous mutants (D, D’) or when *cofilin* is overexpressed (E, E’). (F) Quantification of the number of F-actin aggregates in the anterior portion of the eye discs of the indicated genotypes. Compared to *ey>p35*, the number of F-actin aggregates are significantly (****p < 0.0001) increased in the *ey>hid-p35* discs. This increase is significantly (****p < 0.0001) reduced in response to expression of *dronc*^*RNAi*^, *cofilin* or in *LIMK1*^*2*^ mutants.

LIMK1 is best known to inhibit Cofilin and promote F-actin polymerization. Suppression of AiP by loss of LIMK1 or gain of Cofilin suggests that formation of actin filaments might be important for AiP. We therefore examined the F-actin pattern in larval discs of various genetic backgrounds using phalloidin (PHN), a F-actin specific marker. Compared to the control *ey>p35* (Fig 3A and 3A’), in *ey>hid-p35*, bright F-actin signals with an increased number of actin aggregates were observed in the anterior overgrown part of the disc (arrows and arrowheads, Fig 3B and 3B’, aggregate numbers are quantified in Fig 3F). It has been reported that, in epithelial tissues, apoptotic cells can be extruded basally via mechanisms depending on actin remodeling in neighboring cells [31]. We therefore labeled the *ey>hid-p35* disc with the cleaved caspase-3 antibody, a marker of the activated initiator caspase Dronc in *Drosophila* [9], to indicate cells with high Dronc activities although execution of cell death is blocked by P35 (S2A Fig). Notably, the majority of the aggregates we observed in *ey>hid-p35* discs are not overlapping with or adjacent to the cells labelled with the cleaved caspase-3 antibodies. Moreover, these aggregates frequently localize at the apical cell cortex in the disc suggesting an accumulation of F-actin independent of cell extrusion (arrows and arrowheads, S2B Fig). Nevertheless, this F-actin accumulation including the formation of actin aggregates depends on Dronc, which coordinates apoptosis and AiP, as knockdown of Dronc suppresses it as well as the AiP-dependent tissue overgrowth (Fig 3C, 3C’ and 3F). This suggests that F-actin accumulates downstream of Dronc. As expected, *LIMK1*^*2*^ mutants or overexpression of Cofilin also suppresses this actin accumulation (Fig 3D–3F). To determine whether such effects are tissue-specific, we further examined larval wing imaginal discs by co-expression of *hid* and *p35* under the control of *nub-GAL4*, a wing pouch-specific driver. LIMK1-dependent tissue overgrowth and F-actin accumulation were also observed in wing discs (S3 Fig). Therefore, LIMK1 and Cofilin regulate F-actin dynamics and AiP-dependent tissue overgrowth in both eye and wing epithelial tissues.

**Fig 4:**
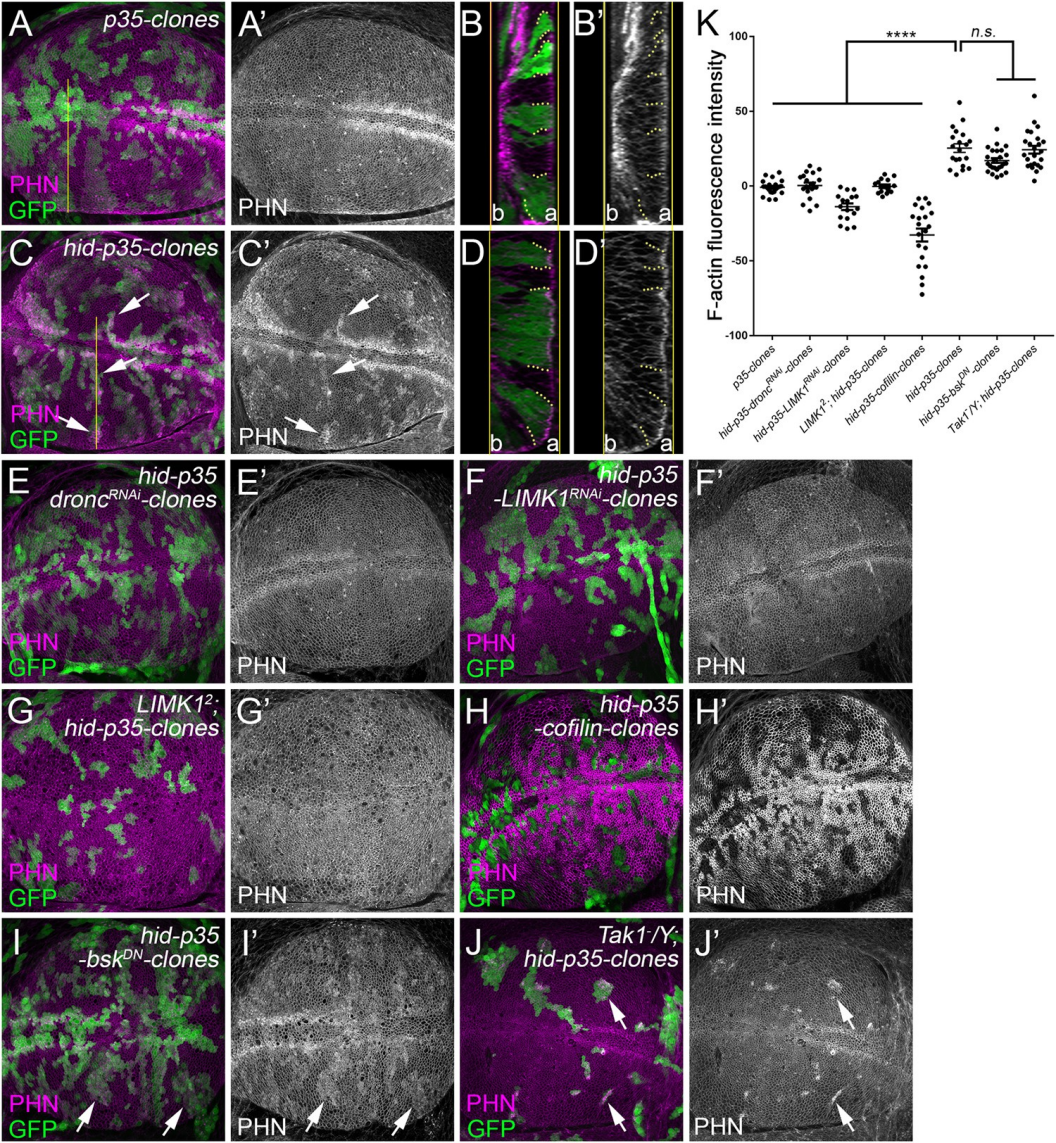
F-actin accumulation in *hid-p35* clones depends on LIMK1 and Cofilin, but not JNK signaling. (A-J’) Late 3^rd^ instar wing discs with 48-hour old mosaic clones positively marked by GFP. Phalloidin (PHN, magenta in A-J and grey in A’-J’) labels F-actin. Images of the apical side of the discs are shown in A, C and E-J. B and D show the cross sections corresponding to the yellow lines indicated in A and C, respectively. The apical (a) and basal (b) surfaces of these cross sections are as indicated. Compared to the control clones expressing P35 only (A-B’), simultaneous expression of *hid* and *p35* (*hid-p35* clones) leads to an accumulation of F-actin apically in the clones (arrows in C, C’ and clones highlighted by yellow dotted lines in D, D’). This F-actin accumulation in *hid-p35* clones is suppressed by expression of *dronc*^*RNAi*^ (E, E’), *LIMK1*^*RNAi*^ (F, F’) or in *LIMK1*^*2*^ hemizygous mutants (G, G’). Expression of *cofilin* in the *hid-p35* clones further reduces the level of F-actin (H, H’). In contrast, F-actin accumulation persists the *hid-p35* clones with expression of a dominant negative mutant of *bsk* (*bsk*^*DN*^, I, I’, arrows) or in a *Tak1* hemizygous null mutant background (J, J’, arrows). (K) Quantification of the F-actin signal intensity in the representative clones of the indicated genotypes in the wing disc pouches. Images of the apical side of the discs were used. Compared to the *p35* clones, the DHE signals are significantly (****p < 0.0001) increased in the *hid-p35* clones. This increase is significantly (****p < 0.0001) reduced in response to expression of *SOD*, *cofilin* or in *LIMK1*^*2*^ mutants. In contrast, expression of *bsk*^*DN*^ or *Duox*^*RNAi*^ has no significant (n.s.) effects.

Considering that the *hid-p35*-expressing discs are overgrown, the actin accumulation we observed in these tissues can potentially be a secondary effect caused by tissue overgrowth. Therefore, in addition to the whole disc analysis, we also employed a clonal analysis to determine whether F-actin accumulates in AiP independently of tissue overgrowth. We co-expressed *hid* and *p35* in heat-shock-induced clones (*hid-p35* clones) and examined F-actin in these clones 48 hours after their induction (Fig 4). These clones do not show any signs of tissue overgrowth. We focused our analysis on the larval wing disc pouch because it is a flat epithelial tissue most suitable for the observation of potential F-actin pattern changes. Compared to the control clones expressing *p35* (Fig 4A–4B’), F-actin accumulates apically and cortically in the *hid-p35* clones (arrows, Fig 4C–4D’, and quantified in Fig 4K). As observed in whole discs, F-actin accumulation in *hid-p35* clones is suppressed by loss of either *dronc* (via RNAi, Fig 4E, 4E’ and 4K) or LIMK1 (via RNAi or mutants, Fig 4F–4G’ and 4K). Notably, loss of *dronc* or *LIMK1* alone does not affect the F-actin organization (S4A-S4C’ Fig). This further confirms that LIMK1-dependent F-actin accumulation occurs downstream of Dronc. Overexpression of Cofilin promotes depolymerization of F-actin. Indeed, it reduces the F-actin levels in both control and *hid-p35* clones although the clones appear to be smaller in size (Figs 4H, 4H’, 4K and S4D and S4D’). Intriguingly, expression of a dominant negative mutant of JNK (*bsk*^*DN*^) does not inhibit the F-actin accumulation in *hid-p35* clones (Fig 4I, 4I’ and 4K). Consistently, this F-actin accumulation also persists in a null mutant of Tak1 (Fig 4J, 4J’ and 4K), an upstream kinase in the JNK pathway critical for AiP [15]. As controls, expression of *bsk*^*DN*^ or the mutant of *Tak1* does not affect the F-actin organization by themselves (S4E-S4F’ Fig). Taken together, these data suggest that F-actin accumulates upstream or in parallel of activation of JNK during AiP.

### F-actin polymerization is required upstream of JNK to induce AiP

**Fig 5:**
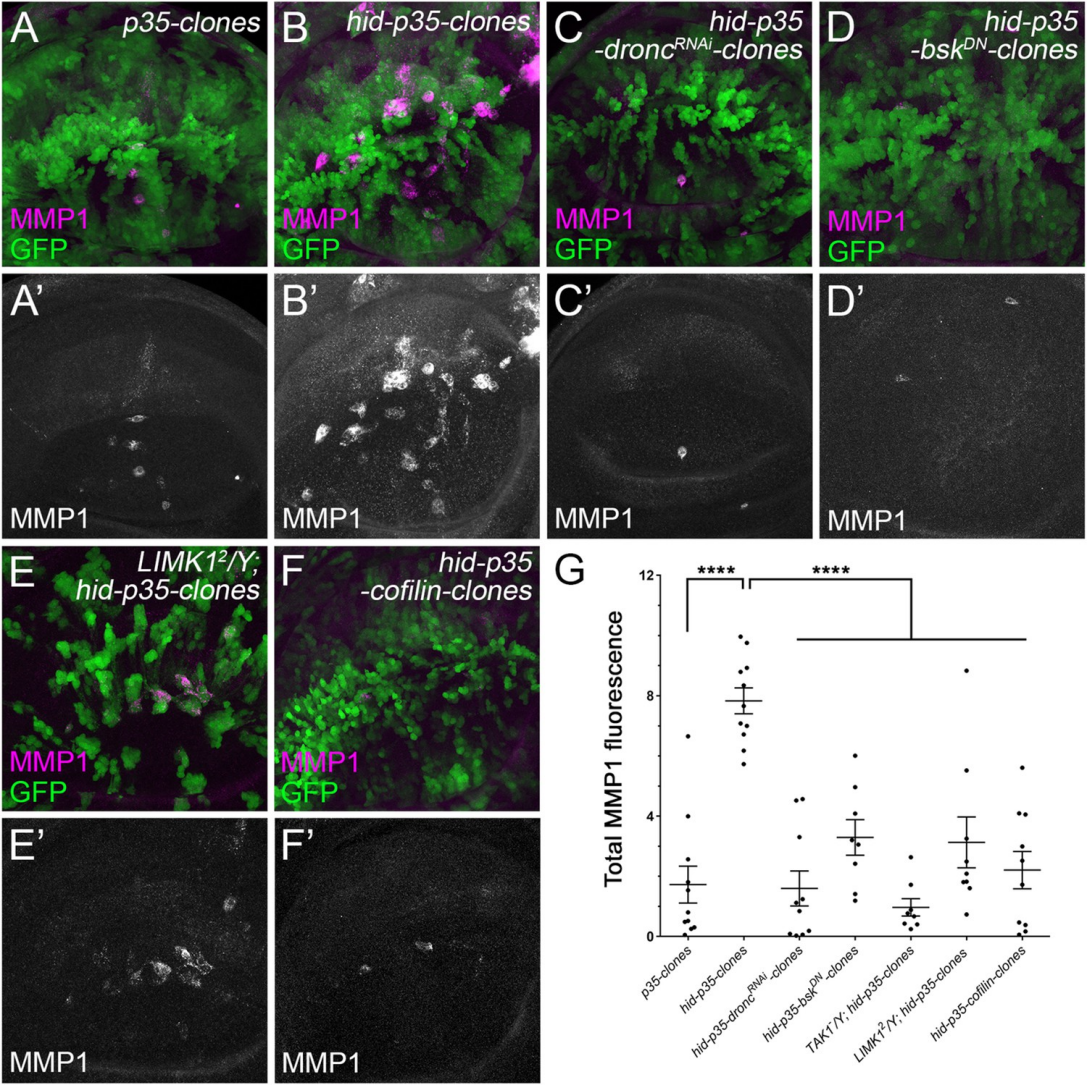
LIMK1 and Cofilin regulate activation of JNK in *hid-p35* clones. (A-F’) Late 3^rd^ instar wing discs with 48-hour old mosaic clones positively marked by GFP. MMP1 (magenta in A-F and grey in A’-F’) is a marker of JNK activity. Images of Z-stack projections are shown as the MMP1 signals localize at the basal part of the disc. A low level of MMP1 signals was detected in the control clones expressing P35 (A, A’). In contrast, a strong increase of MMP1 labelling was observed in the clones simultaneously expressing *hid* and *p35* (*hid-p35* clones) (B, B’). Such a strong level of MMP1 signals is largely inhibited by expression of *dronc*^*RNAi*^ (C, C’), *bsk*^*DN*^ (D, D’), or in *LIMK1*^*2*^ mutants (E, E’). Expression of *cofilin* also suppresses the *hid-p35*-induced MMP1 expression (F, F’). (G) Quantification of the MMP1 signal intensity in the representative wing disc pouches carrying clones of the indicated genotypes. Compared to the discs with *p35* clones, the MMP1 signals are significantly (****p < 0.0001) increased in the discs with *hid-p35* clones. This increase is significantly (****p < 0.0001) reduced in response to expression of *dronc*^*RNAi*^, *bsk*^*DN*^, *cofilin* or in *Tak1* hemizygous null mutants or *LIMK1*^*2*^ mutants.

To further determine the relationship between F-actin polymerization and JNK activation in AiP, we examined the expression level of MMP1, a downstream target of JNK using clonal analysis. Compared to the control clones (Fig 5A and 5A’), expression of MMP1 is strongly induced in *hid-p35* clones (Fig 5B and 5B’, quantified in Fig 5G). As expected, such an increase of MMP1 is suppressed by knockdown of *dronc*, expression of *bsk*^*DN*^ or a null mutant of Tak1 (Fig 5C, 5D’ and 5G). Notably, the increased MMP1-labelling in *hid-p35* clones is also strongly suppressed in the *LIMK1*^*2*^ mutants or by expression of Cofilin (Fig 5E-5G). This was further confirmed in the whole eye discs using TRE-red, another reporter of JNK activity [32]. Expression of *LIMK1 RNAi* or Cofilin suppresses the activation of JNK in *ey>hid-p35* eye discs (S5 Fig). Therefore, the F-actin polymerization, regulated by LIMK1 and Cofilin, is required for activation of JNK, the critical step triggering AiP.

### F-actin polymerization is required for ROS production upstream of JNK

**Fig 6:**
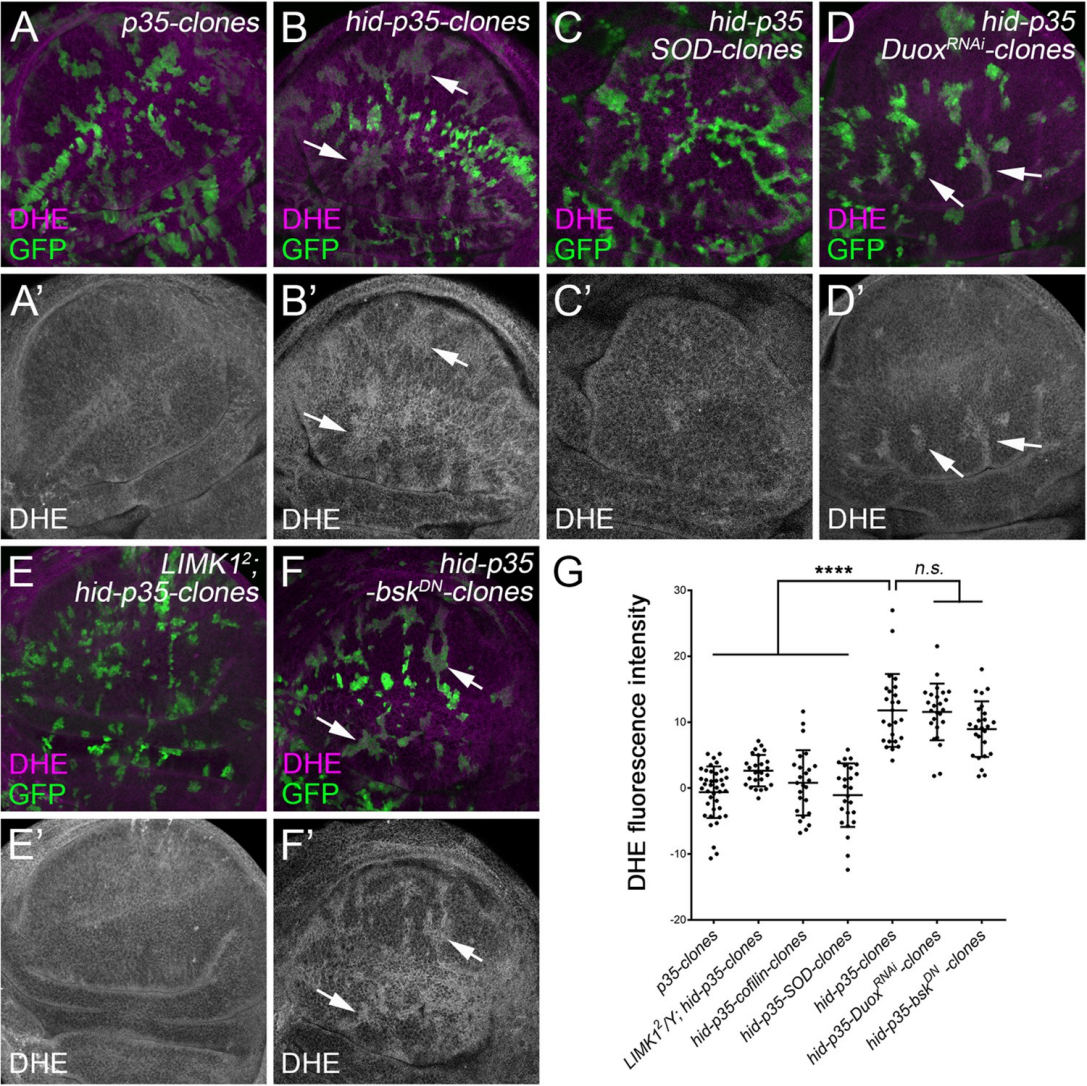
LIMK1 and Cofilin regulate production of cytosolic ROS in *hid-p35* clones. (A-F’) Late 3^rd^ instar wing discs with 48-hour old mosaic clones positively marked by GFP. Dihydroethidium (DHE) staining (magenta in A-F and grey in A’-F’) is used to indicate the level of ROS. Images of the apical side of the discs are shown. Compared to the control clones expressing P35 only (A, A’), simultaneous expression of *hid* and *p35* (*hid-p35* clones) leads to an increased production of ROS in the clones (B, B’, arrows). This increased ROS production is suppressed by expression of SOD, a superoxide dismutase (C, C’). In contrast, RNAi knockdown of Duox, a transmembrane NADPH oxidase, does not obviously affect the level of ROS in *hid-p35* clones (D, D’, arrows). Increased ROS production in *hid-p35* clones is also inhibited in a *LIMK1*^*2*^ mutant background (E, E’), but not in response to expression of *bsk*^*DN*^, (F, F’, arrows). (G) Quantification of the DHE signal intensity in the representative clones of the indicated genotypes in the wing disc pouches. Compared to the *p35* clones, the DHE signals are significantly (****p < 0.0001) increased in the *hid-p35* clones. This increase is significantly (****p < 0.0001) reduced in response to expression of *SOD*, *cofilin* or in *LIMK1*^*2*^ mutants, but not (n.s.) in response to expression of *bsk*^*DN*^ or *Duox*^*RNAi*^.

ROS production has recently been reported to mediate activation of JNK in AiP [19, 21]. We therefore investigated whether F-actin polymerization is required for ROS production using clonal analysis. Compared to the control clones expressing P35 (Fig 6A and 6A’), ROS, indicated by Dihydroethidium (DHE) staining, increase in *hid-p35* clones (arrows, Fig 6B and 6B’, quantified in Fig 6G). Expression of SOD, a superoxide dismutase reducing cytosolic ROS, in *hid-p35* clones suppresses the clonal increase of ROS (Fig 6C, 6C’ and 6G) as well as activation of JNK (S6A and S6B Fig). Duox, a transmembrane NADPH oxidase, has been reported to produce extracellular ROS and attract hemocytes to promote AiP and tissue overgrowth of the whole eye discs [21]. However, RNAi knockdown of Duox does not significantly inhibit ROS production in *hid-p35* clones (Fig 6D, 6D’ and 6G). The *SOD* and *Duox*^*RNAi*^ experiments suggest that, in *hid-p35* clones, largely cytosolic ROS are produced. Consistently, unlike the whole eye discs where extracellular ROS recruit hemocytes for the AiP-dependent tissue overgrowth [21, 22], hemocytes were rarely observed to be associated with wing discs containing *hid-p35* clones (S6C–S6D’ Fig). Importantly, the increase of ROS in *hid-p35* clones is completely suppressed in the *LIMK1*^*2*^ mutants or by expression of Cofilin (Fig 6E, 6E’ and 6G). In contrast, these ROS are not affected by expression of *bsk*^*DN*^ (Fig 6F–6G). This is consistent with the published data that ROS act upstream of JNK [18, 21]. Altogether, our data suggest that LIMK1- and Cofilin-dependent F-actin polymerization is required for ROS production upstream of JNK during AiP.

### Dronc and LIMK1 genetically interact and drive F-actin polymerization via Myo1D

**Fig 7:**
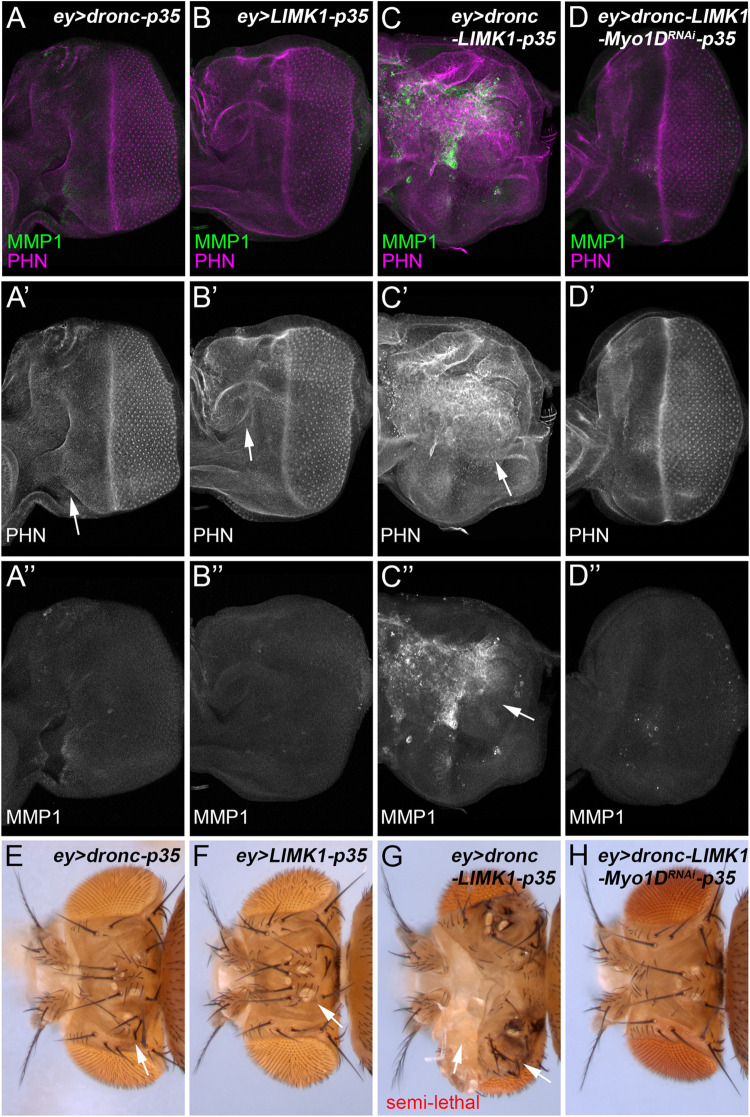
Dronc and LIMK1 drive F-actin polymerization and tissue overgrowth via Myo1D. (A-D”) Late 3^rd^ instar eye discs labelled with PHN (magenta in A-D and grey in A’-D’) and MMP1 (green in A-D and grey in A”-D”). Expression of *dronc* (A, A’) or *LIMK1* in *ey>p35* discs results in a moderate F-actin accumulation as indicated by the PHN labelling and a slight tissue overgrowth (A-B’, arrows). No obvious MMP1 signals are induced in these genetic backgrounds (A”, B”). In contrast, co-expression of *dronc* and *LIMK1* induces a strong accumulation of F-actin and a massive overgrowth of *ey>p35* discs (C, C’, arrow). These are associated with a strong increase of MMP1 signals (C”, arrow). Such tissue overgrowth, increase of F-actin accumulation and MMP1 signals are suppressed by an RNAi knockdown of Myo1D (D-D”), an unconventional myosin. (E-H) Representative adult fly head images of the indicated genotypes. Expression of *dronc* (E) or *LIMK1* (F) in *ey>p35* causes weak to moderate head capsule phenotypes indicated by a duplication of bristles and/or ocelli (E, F, arrows). In contrast, co-expression of *dronc* and *LIMK1* in *ey>p35* results in semi-lethality with escapers showing severe head phenotypes, indicated by deformed head capsules with outgrown and amorphic tissues (G, arrows). These phenotypes are completely suppressed by expression of *Myo1D*^*RNAi*^ (H).

To further determine how F-actin dynamics is regulated in response to apoptotic stress, we examined the relationship between LIMK1 and Dronc, the caspase activating AiP in the proliferating eye and wing tissues [3, 33]. Overexpression of Dronc or LIMK1 alone induces a moderate overgrowth of *ey>p35* eye discs (Fig 7A–7B’, compared to Fig 3A), which is associated with a weak level of F-actin accumulation and JNK activation (Fig 7A” and 7B”). In contrast, co-expression of both Dronc and LIMK1 triggers a strong F-actin accumulation, JNK activation and tissue overgrowth (Fig 7C–7C”). These data suggest that Dronc and LIMK1 genetically interact.

Myo1D, an unconventional myosin, has been previously shown to physically interact with Dronc and mediate its role on activation of JNK in AiP [20]. We therefore examined whether Myo1D mediates the interaction between Dronc and LIMK1. RNAi knockdown of Myo1D completely suppresses the phenotypes of F-actin accumulation, JNK activation and tissue overgrowth induced by co-expression of Dronc and LIMK1 (Fig 7D–7D”). Consistent with this, expression of Dronc or LIMK1 alone results in adult heads with moderate overgrowth phenotypes, including duplicated ocelli and bristles (Fig 7E and 7F). In contrast, co-expression of Dronc and LIMK1 causes semi-lethality with escapers showing severe adult head overgrowth phenotype (Fig 7G). This overgrowth phenotype is completely suppressed by knockdown of Myo1D (Fig 7H). These suggest that Myo1D mediates the synergistic roles of Dronc and LIMK1 on promoting F-actin polymerization and tissue overgrowth.

**Fig 8:**
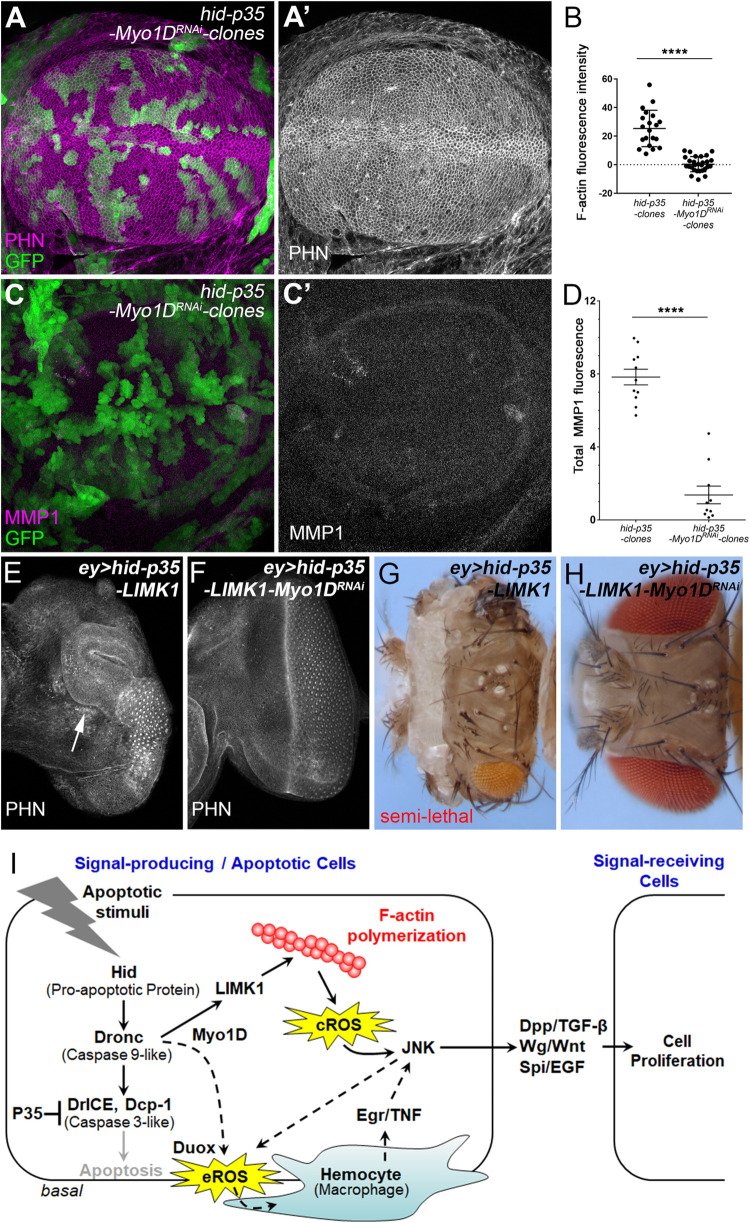
Dronc and LIMK1 drive F-actin polymerization and tissue overgrowth via Myo1D. (A, A’) Apical side images of a late 3^rd^ instar wing discs with 48-hour old mosaic clones, positively marked by GFP and labelled with PHN (magenta in A and grey in A’). Expression of *Myo1D*^*RNAi*^ suppresses F-actin accumulation in *hid-p35* clones (compared to Fig 4C and 4C’). (B) Quantification of the F-actin signal intensity in the representative clones of the indicated genotypes in the wing disc pouches. Compared to the *hid-p35* clones, expression of *Myo1D*^*RNAi*^ significantly (****p < 0.0001) reduces F-actin levels in *hid-p35* clones. (C, C’) Images of Z-stack projections of a late 3^rd^ instar wing discs with 48-hour old mosaic clones, positively marked by GFP and labelled with MMP1 (magenta in C and grey in C’). Expression of *Myo1D*^*RNAi*^ reduces the MMP1 level in *hid-p35* clones (compared to Fig 5B and 5B’). (D) Quantification of the MMP1 signal intensity in the representative wing disc pouches with clones of the indicated genotypes. Expression of *Myo1D*^*RNAi*^ significantly (****p < 0.0001) reduces the MMP1 levels in the wing disc pouches carrying *hid-p35* clones. (E, F) Late 3^rd^ instar eye discs of the indicated genotypes labelled with PHN. Expression of LIMK1 enhances the overgrowth phenotype in *ey>hid-p35* by inducing further tissue outgrowth (E, arrow). This overgrowth phenotype is suppressed by expression of *Myo1D*^*RNAi*^ (F). (G, H) Representative adult fly head images of the indicated genotypes. Expression of LIMK1 in *ey>hid-p35* results in semi-lethality with escapers all showing severe overgrowth of head capsules and loss of eye tissues (G). These phenotypes are completely suppressed by expression of *Myo1D*^*RNAi*^ (H). (I) Model summarizing roles of F-actin polymerization and its regulation in AiP. The data presented in this paper suggested that F-actin polymerization occurs in apoptotic signal-producing cells and is required for production of the cytosolic ROS (cROS) and its subsequent activation of JNK leading to AiP via secreting growth signals such as Dpp/TGF-β, Wg/Wnt and Spi/EGF. In response to apoptotic stress, Dronc may interact with LIMK1 via Myo1D to trigger F-actin polymerization and the initial activation of JNK. Moreover, Myo1D also mediates activation of Duox and production of the extracellular ROS (eROS) [21]. eROS work together with JNK to disrupt the basement membrane and attract hemocytes, the *Drosophila* macrophages, to further promote JNK activation non-cell autonomously via secretion of the ligand Eiger (Egr)/TNF [20, 22]. We propose that these mechanisms, indicated by the dashed arrows, amplifies LIMK1-mediated JNK activation leading to tissue overgrowth.

Furthermore, we examined the roles of Myo1D in F-actin remodeling during AiP by clonal analysis. RNAi knockdown of Myo1D inhibits F-actin accumulation and its downstream activation of JNK in *hid-p35* clones (Fig 8A-8D). Moreover, consistent with the role of LIMK1 in promoting F-actin polymerization and AiP, overexpression of LIMK1 enhances the *ey>hid-p35* overgrowth phenotype indicated by an extensive tissue outgrowth (arrow, Fig 8E, compared to Figs 3B and S2A’). This results in adult semi-lethality with escapers showing severe head overgrowth phenotype with mostly loss of eyes (Fig 8G, compared to Fig 1C–1E). This enhanced phenotype is suppressed by expression of *Myo1D*^*RNAi*^ (Fig 8F and 8H). Taken together, these data further confirm the critical role of Myo1D in mediating LIMK1-dependent F-actin polymerization and activation of AiP (Fig 8I).

## Discussion

This study revealed an accumulation of F-actin in response to apoptotic stress and its essential role in activation of AiP (Fig 8I). LIMK1, an evolutionary conserved kinase promoting F-actin polymerization via phosphorylating and inhibiting Cofilin, acts as a key regulator of AiP. Our results further suggest that such F-actin remodeling depends on the initiator caspase Dronc and the unconventional myosin Myo1D. Importantly, actin polymerization mediates production of cytosolic ROS (cROS) and activation of JNK, which trigger AiP. Therefore, the dynamic behavior of actin filaments appears to be critical for the communication between apoptotic cells and their neighbors.

As the key structural network of the cell, the actin cytoskeleton is highly dynamic to support various cell behaviors including growth, proliferation, migration, and death

[24, 25]. It is therefore regulated both spatially and temporally to mediate the cellular responses to internal or external signals. For example, actin cytoskeleton reorganization is essential for the characteristic morphological changes during apoptosis such as cell shrinkage and plasma membrane blebbing [34, 35]. In mammalian cells, effector caspases including caspase-3 were found to cleave cytoskeletal actin directly or Gelsolin, an actin-binding protein, to promote F-actin depolymerization and apoptosis [36–38]. Caspase-3 can also cleave and activate ROCK1, a kinase acting on cytoskeleton, leading to membrane blebbing [39]. Furthermore, in epithelial tissues *in vivo*, apoptotic cells are frequently extruded leading to delamination, a process depending on actin accumulation in the neighboring cells [31]. Therefore, actin remodeling is critical for execution of apoptosis. Interestingly, actin reorganization has also been observed at the induction stage of apoptosis. A recent study using time-lapse imaging detected an early accumulation of F-actin apically in irradiation-induced apoptotic cells in the *Drosophila* developing wing epithelium, prior to the basal extrusion of these cells and the appearance of any noticeable apoptotic characteristics such as cell shrinkage, nuclear condensation, and fragmentation [40]. The exact role of this early actin accumulation is not yet known. In this study, we have observed a similar actin accumulation in the apoptotic cells prior to the execution of cell death. This F-actin polymerization occurs downstream of the initiator caspase Dronc. It appears to be an early cellular response to the apoptotic stress as it mediates ROS production and JNK activation, which act as signals to communicate with the neighboring cells and trigger AiP. Notably, unlike stress-induced apoptosis, developmental apoptosis does not induce AiP although the core apoptosis machineries employed in both situations are the same. It is currently unknown what differentiates these two types of apoptosis leading to distinct responses of AiP. The answer may lie in the context-dependent actin dynamics. It would be interesting to further examine and compare the F-actin behaviors in stress-induced *versus* developmental apoptosis.

Another intriguing question raised from our study is how the apoptotic stress promotes F-actin polymerization. We showed that the initiator caspase Dronc is essential for this process. It genetically interacts with LIMK1 leading to actin polymerization, JNK activation and tissue overgrowth when execution of apoptosis is blocked (Fig 7). Notably, the human LIMK1 can be cleaved by caspase-3 and become constitutively active to promote membrane blebbing in apoptotic cells in culture [41]. This is unlikely the case in our study because 1) AiP in the proliferating tissues depends on the initiator caspase Dronc, but not the effector caspases; and 2) F-actin polymerization observed in AiP occurs at an early stage of the apoptotic response, prior to any noticeable cellular morphological changes. However, it is still possible that LIMK1 is cleaved and activated by Dronc because the catalytic activity of Dronc is believed to be required for AiP [3, 15]. Alternatively, LIMK1 may be involved in AiP without being cleaved as its expression is increased when AiP occurs (Fig 1Q). The mechanisms regulating expression of LIMK1 and its activity therefore deserve to be further investigated.

In this study, we also observed that the interaction between Dronc and LIMK1 is mediated by Myo1D. Loss-of-Myo1D inhibits actin polymerization, JNK activation, and, consequently, induction of AiP (Fig 8). We have previously shown that, during AiP, Dronc relocates from cytoplasm to plasma membrane via a physical interaction with Myo1D [20]. It is possible that LIMK1-dependent F-actin polymerization assists the subcellular localization of Dronc to promote AiP. Notably, Myo1D also mediates a role of Dronc in activation of Duox, a membrane-bound NADPH oxidase, to produce extracellular ROS and attract hemocytes which in turn secretes Eiger, the ligand to activate JNK non-cell autonomously [20, 21]. This process is important for AiP-dependent tissue overgrowth as loss-of-Duox or expression of scavengers specifically targeting the extracellular ROS suppresses overgrowth of the *ey>hid-p35* discs [21]. Interestingly, in our clonal analysis of AiP which does not induce tissue overgrowth, it is the cytosolic ROS, but not the extracellular ROS produced by Duox, triggering action of JNK (Fig 6). In this case, although the mechanism controlling the cytosolic ROS production is unknown, JNK is not required because blocking JNK has no effects. However, in overgrown *ey>hid-p35* discs, JNK, together with the extracellular ROS, damages the epithelial basement membrane and recruit hemocytes to promote tissue overgrowth [22]. Moreover, it has been reported that JNK can activate a feedback amplification loop via regulation of pro-apoptotic genes such as *hid* and *rpr* in apoptotic cells [42]. Therefore, it is possible that the cytosolic ROS are required for the initial activation of JNK in apoptotic cells to trigger AiP. When these apoptotic cells are kept ‘undead’, activated JNK promotes and works together with production of ROS including the extracellular ROS to recruit hemocytes and further amplify the JNK activity leading to tissue overgrowth (Fig 8I). Further investigation of the underlying mechanisms is required to test this hypothesis.

## Material and methods

### *Drosophila* genetics and stocks

Genetic crosses for all experiments were reared at 25°C unless otherwise noted. Two *ey>hid-p35* stocks were used to analyze AiP-dependent tissue overgrowth phenotypes. Their exact genotypes are *UAS-hid; ey-Gal4 UAS-p35*/*CyO*,*tub-Gal80* and *UAS-hid; ey-Gal4 UAS-p35*/*TM6B*,*tub-Gal80*. The stock *UAS-hid; ey-Gal4 UAS-p35*/*CyO*,*tub-Gal80* was used to cross with mutants or transgenic lines to identify potential AiP regulators by scoring the adult hyperplastic overgrowth phenotypes of the F1 offspring. These phenotypes were categorized into three groups, weak (W), moderate (M) and severe (S), as previously described [15]. The stock *UAS-hid; ey-Gal4 UAS-p35*/*TM6B*,*tub-Gal80* was used for the analyses of larval eye discs because of the convenience to identify the correct larval genotypes. Accordingly, the *ey>p35* controls used in each experiment are the *ey-Gal4 UAS-p35* on either second or third chromosome as appropriate.

*UAS-LIMK1*^*RNAi*^ lines (26294, 42576 and 57012), *LIMK1*^*2*^ (59033), *UAS-LIMK1* (9117), *UAS-cofilin* (9235), *UAS-dronc*^*RNAi*^ (32963), *UAS-Myo1D*^*RNAi*^ (33971), *UAS-SOD* (24750), *UAS-mCherry* (38424), *T80-GAL4* (1878), *nub-GAL4* (86108), *TAK1*^*2527*^ (58809) and *TRE-red* (59012) were obtained from the Bloomington *Drosophila* Stock Center. *Dorsal Eye-Gal4* (*DE-Gal4*) [43], *UAS-bsk*^*DN*^ [44], *UAS-dronc* [45], *UAS-Duox*^*RNAi*^ [46] and *GMR-hid* [47] are as described.

### *DE*^*ts*^*>hid* regeneration assay

Larvae of the following genotypes 1. *DE*^*ts*^*>hid* (*UAS-hid/+; UAS-GFP/+; DE-Gal4 tub-Gal80*^*ts*^*/+*)*; 2*. *DE*^*ts*^*>LIMK1*^*RNAi*^ (*UAS-GFP/+; DE-Gal4 tub-Gal80*^*ts*^*/UAS-LIMK1*^*RNAi-57012*^); 3. *DE*^*ts*^*>hid-LIMK1*^*RNAi*^ (*UAS-hid/+; UAS-GFP/+; DE-Gal4 tub-Gal80*^*ts*^*/UAS-LIMK1*^*RNAi-57012*^)*; 4*. *DE*^*ts*^*>cofilin* (*UAS-GFP/+; DE-Gal4 tub-Gal80*^*ts*^*/UAS-cofilin*); 5. *DE*^*ts*^*>hid-cofilin* (*UAS-hid/+; UAS-GFP/+; DE-Gal4 tub-Gal80*^*ts*^*/UAS-cofilin*) were raised at 18°C. Expression of the UAS-constructs (*GFP*, *hid*, *LIMK1*^*RNAi*^, *cofilin*) was induced by a temporal temperature shift to 30°C for 12 hours. This was then followed by a 72 h recovery period at 18°C before the late 3^rd^ instar eye discs of each genotype were dissected and analyzed. Full details of the *DE*^*ts*^*>hid* assay have been described previously [15].

### Mosaic analysis

For mosaic analysis with clones expressing transgenes, a heat shock inducible FLP-out assay combined with the GAL4-UAS system was used [48, 49]. Late second instar (64-72h post-hatching) larvae of the following genotype were heat shocked at 37°C for 7 minutes and then grew at 25°C for 48 hours before they were dissected and analyzed at the late 3^rd^ instar stage. (1) Generation of the control in larval *p35* expressing clones: *hs-FLP/+; act>y*^*+*^*>GAL4 UAS-GFP/+; UAS-p35/+*. (2) Generation of *hid* and *p35* co-expressing clones: *hs-FLP/UAS-hid; act>y*^*+*^*>GAL4 UAS-GFP/+; UAS-p35/+*. (3) Generation of clones expressing *hid*, *p35* and *dronc*^*RNAi*^: *hs-FLP/UAS-hid; act>y*^*+*^*>GAL4 UAS-GFP/+; UAS-p35/UAS-dronc*^*RNAi*^. (4) Generation of clones expressing *hid*, *p35* and *LIMK1*^*RNAi*^: *hs-FLP/UAS-hid; act>y*^*+*^*>GAL4 UAS-GFP/+; UAS-p35/UAS-LIMK1*^*RNAi-57012*^. (5) Generation of clones expressing *hid*, *p35* and *cofilin*: *hs-FLP/UAS-hid; act>y*^*+*^*>GAL4 UAS-GFP/+; UAS-p35/UAS-cofilin*. (6) Generation of clones expressing *hid*, *p35* and *bsk*^*DN*^: *hs-FLP/UAS-hid; act>y*^*+*^*>GAL4 UAS-GFP/+; UAS-p35/UAS-bsk*^*DN*^. (7) Generation of clones expressing *hid*, *p35* and *SOD*: *hs-FLP/UAS-hid; act>y*^*+*^*>GAL4 UAS-GFP/+; UAS-p35/UAS-SOD*. (8) Generation of clones expressing *hid*, *p35* and *Duox*^*RNAi*^: *hs-FLP/UAS-hid; act>y*^*+*^*>GAL4 UAS-GFP/UAS-Duox*^*RNAi*^*; UAS-p35/+*. (9) Generation of clones expressing *hid*, *p35* and *Myo1D*^*RNAi*^: *hs-FLP/UAS-hid; act>y*^*+*^*>GAL4 UAS-GFP/+; UAS-p35/UAS-Myo1D*^*RNAi*^. For mosaic analysis with *hid-p35* clones in the *LIMK* or *TAK1* hemizygous mutant background, late second instar larvae of the following genotype were heat shocked at 37°C for 1 hour and then grew at 25°C for 48 hours before they were dissected and analyzed: (1) *LIMK*^*2*^*/Y; act>y*^*+*^*>GAL4 UAS-GFP/+; UAS-hid UAS-p35/hs-FLP* and (2) *TAK1*^*2527*^*/Y; act>y*^*+*^*>GAL4 UAS-GFP/+; UAS-hid UAS-p35/hs-FLP*. The same *act>y*^*+*^*>GAL4* FLP-out assay was used to generate control clones without expression of *hid* and *p35*.

### Immunolabeling, phalloidin and DHE stainings

Late 3^rd^ instar larval eye and wing discs were dissected, fixed (with 4% paraformaldehyde for 30min at room temperature), and then labeled with antibodies using standard protocols as described [50]. Primary antibodies used were rabbit anti-PH3 (1:1000, Merck 06–570), rabbit anti-cDcp1 (the cleaved Dcp-1 antibody, 1:500, Cell Signaling 9578), rabbit anti-cCasp3 (the cleaved caspase-3 antibody, 1:500, Cell Signaling 9661), rat anti-ELAV (1:50, DHSB 7E8A10), mouse anti-MMP1 (all 1:50, DHSB 3A6B4, 3B8D12 and 5H7B1 used as a 1:1:1 cocktail) and mouse anti-Hemese (H2, 1:20, a gift from István Andó) [51]. Secondary antibodies were goat Fab fragments conjugated to Alex488, 555, or 647 (all 1:1000) from Molecular Probes. Phalloidin (PHN, 1:40, Invitrogen R415) staining of the larval discs was done at room temperature for one hour or together with secondary antibodies at 4°C overnight. Dihydroethidium (DHE, Invitrogen D23107) staining was done as previously described [21].

### Quantitative reverse transcription PCR (RT-qPCR)

To measure expression of *LIMK1* and *cofilin* in *ey>hid-p35* (*UAS-hid;; ey-Gal4 UAS-p35*/*+*) *versus* the control *ey>p35-mCherry* (*ey-Gal4 UAS-p35*/*UAS-mCherry*), 100 third instar larval eye discs of each genotype were used to extract total RNA with an RNeasy Plus Mini Kit (Qiagen). For the analysis of LIMK1 RNAi efficiency, *T80-GAL4* was used to drive expression of *LIMK1*^*RNAi*^, 26294 or 57012, ubiquitously in all 3^rd^ instar larval imaginal discs. Total RNA was then isolated from 100 imaginal discs collected from either the control *T80-GAL4* or *T80-GAL4 UAS-LIMK1*^*RNAi*^ 3^rd^ instar larvae using the TRI Reagent Solution (Invitrogen). Following RNA extraction, cDNA was generated from 300ng of total RNA with the GoScript Reverse Transcription System (Promega) for each genotype. This was followed by the real-time PCR using the SensiFAST SYBR Hi-Rox kit (Bioline) with an ABI Prism7000 system (Life Technologies). *LIMK1* or *cofilin* mRNA levels were normalized to the reference gene *ribosomal protein L32* (*RPL32*) by using the ΔΔCt analysis. Three independent biological repeats were analyzed in triplicate for each experiment. The following primers suggested by the FlyPrimerBank [52] were used: *LIMK1* Fw, GTGAACGGCACACCAGTTAGT; *LIMK1* Rv, ACTTGCACCGGATCATGCTC; *RPL32* Fw, AGCATACAGGCCCAAGATCG; *RPL32* Rv, TGTTGTCGATACCCTTGGGC. The primers used for *cofilin* were Fw, GTGAAAGAAGGCGGAAGGTTAA, and Rv, CACAGTTACACCAGAAGCCATT, as described [53].

### Imaging, quantification and statistical analysis

Adult fly eye images were taken using a Zeiss stereomicroscope equipped with an AxioCam ICC1 camera. Fluorescent eye and wing disc images were taken with a Zeiss LSM 710 confocal microscope. For quantification of the number of PH3-positive cells (Fig 1P) and F-actin aggregates (Fig 3F), ImageJ with the cell counter plugin was used to score representative larval eye discs (n≥10) from each genotype. For quantification of the fluorescence intensity of PHN labeling (Figs 4K and 8B), MMP1 labeling (Figs 5G, 8D and S6B) or DHE staining (Fig 6G) in mosaic analysis and TRE-red signals in larval eye discs (S5F Fig), ImageJ was used to measure the fluorescence intensity of the area of interest (ROI) and its corresponding background area in each representative disc to calculate their mean value difference as the normalized fluorescence intensity of ROI. The ROIs and their background areas measured were selected using drawing tools in discs as follows: (1) to measure PHN or DHE signals, ROIs are the representative clones (n≥15) in wing disc pouches while the background is their adjacent non-clonal areas; (2) to measure the MMP1 signals, ROIs are the whole wing disc pouches (n≥8) while the background is the non-clonal areas in the same wing pouches; and (3) to measure the TRE-red signals, ROIs are the whole eye discs (n≥10) while the background is the antenna disc areas without TRE-red signals. For the *DE*^*ts*^*>hid* regeneration assay, at least 10 representative eye discs from each genotype were measured for their sizes of dorsal versus ventral half of discs using the “histogram” function in Adobe Photoshop CS6, as previously described [54]. The statistical analysis in all experiments was conducted with GraphPad Prism 7 using either an unpaired student’s T test (Figs 1Q, 1R, 8B, 8D and S6B) or a one-way ANOVA with Dunnett’s multiple comparison test and plotted with Mean ± SEM.

## Supporting information

S1 Fig LIMK1 and Cofilin do not regulate *hid*-induced apoptosis. (A-D) Late 3^rd^ instar eye discs expressing a *GMR-hid* transgene to induce apoptosis, anterior is to the left. *DE-GAL4* was used to drive expression of various *UAS*-constructs in these discs. The cleaved Dcp-1 (cDcp1) antibodies label apoptotic cells. *GMR-hid* induces two waves of apoptosis as shown in the control (A, arrowheads). Expression of *dronc*^*RNAi*^ under the control of *DE-GAL4* suppresses apoptosis in the dorsal half of the disc (B, highlighted by the yellow dotted line). In contrast, expression of *LIMK1*^*RNAi*^ (C) or *cofilin* (D) does not affect *GMR-hid*-induced apoptosis. (E-H) Representative adult fly eye images of the indicated genotypes. *GMR-hid* induces an eye ablation phenotype as shown in the control (E). Expression of *dronc*^*RNAi*^ driven by *DE-GAL4* partially rescues the dorsal part of the eye due to its suppression of *GMR-hid*-induced apoptosis in this region (F, highlighted by the yellow dotted line). However, expression of *LIMK1*^*RNAi*^ (G) or *cofilin* (H) does not suppress *GMR-hid*-induced eye ablation phenotype.
(TIF)

S2 Fig F-actin aggregates are not associated with Dronc activation and localize apically in *ey>hid-p35* discs. A late 3^rd^ instar *ey>hid-p35* disc labelled with PHN (magenta in A, B and grey in A’, B’) and the cleaved caspase-3 antibodies (cCasp3, green in A and grey in A”), a marker of Dronc activity. B and B’ are enlarged images of the outlined area in A, which are shown together with their vertical and horizontal cross sections on right and bottom, respectively. The apical (a) and basal (b) surfaces of these cross sections are as indicated. Four representative F-actin aggregates on these images are indicated by arrows and arrowheads. F-actin accumulation and aggregates are present in the proliferating portion of the disc anterior to the MF (A, A’). Despite a strong activation of Dronc in the same tissue (A, A”), majority of the F-actin aggregates are not overlapping with or adjacent to the cCasp3-positive cells (B). Moreover, these F-actin aggregates localize apically in the disc (arrows and arrowheads in B and B’).
(TIF)

S3 Fig LIMK1-dependent F-actin accumulation occurs in the *nub>hid-p35* wing discs. (A-C) Late 3^rd^ instar wing discs labelled with PHN. Anterior is to the left. Compared to the control *nub>p35* (A), a massive increase of F-actin filaments was observed in the overgrown wing pouch area of the *nub>hid-p35* discs (B, arrows). Such F-actin accumulation and tissue overgrowth were suppressed by a knockdown of *LIMK1* with RNAi (C).
(TIF)

S4 Fig The F-actin pattern in wing discs is not affected by manipulating the levels of Dronc, LIMK1, Cofilin and JNK. Late 3^rd^ instar wing discs with 48-hour old mosaic clones positively marked by GFP. PHN (magenta in A-F and grey in A’-F’) labels F-actin. Images of the apical part of the discs are shown. The clones expressing *dronc*^*RNAi*^ (A, A’), *LIMK1*^*RNAi*^ (B, B’), or *bsk*^*DN*^ (E, E’) have no effect on the cortical F-actin pattern in the discs. Similarly, the wildtype clones in the *LIMK1*^*2*^ (C, C’) or *Tak1* (F, F’) mutant background does not alter the F-actin pattern either. Notably, expression of *cofilin* (D, D’) reduces the level of F-actin.
(TIF)

S5 Fig LIMK1 and Cofilin regulate activation of JNK in ey>*hid-p35* discs. (A-E’) Late 3^rd^ instar eye discs, anterior is to the left. *TRE-red* (magenta in A-E and grey in A’-E’) is a marker of JNK activity. ELAV (green in A-E) labels photoreceptor neurons therefore indicates the posterior differentiating portion of the eye discs. Compared to the control *ey>p35* (A, A’), *TRE-red* signals strongly increase in the *ey>hid-p35* eye discs (B, B’). This increase is suppressed by expressing *dronc*^*RNAi*^ (C, C’), *LIMK1*^*RNAi*^ (D, D’) or *cofilin* (E, E’). (F) Quantification of the *TRE-red* signal intensity in the eye discs of the indicated genotypes. Compared to the control *ey>p35*, the *TRE-red* signals are significantly (***p < 0.001) increased in the *ey>hid-p35* discs. This increase is significantly (***p < 0.001) reduced in response to expression of *dronc*^*RNAi*^, *LIMK1*^*RNAi*^ or *cofilin*.
(TIF)

S6 Fig Cytosolic ROS, but not hemocytes, are required for JNK activation in *hid-p35* clones. (A-A’) Late 3^rd^ instar wing discs with mosaic clones positively marked by GFP. *hid*, *p35* and *SOD* are simultaneously expressed in the clones for 48 hours. The JNK activity, indicated by the MMP1 labeling (magenta in A and grey in A’), is very low in these clones. (B) Quantification of the MMP1 signal intensity in the representative wing disc pouches carrying clones of the indicated genotypes. Compared to the discs with *hid-p35* clones, the MMP1 signals are significantly (****p < 0.0001) reduced in response to expression of SOD in the clones. (C-D’) Late 3^rd^ instar wing discs with either *p35*-expressing (C, C’) or *hid-p35-*expressing (D, D’) clones positively marked by GFP. Anti-Hemese (H2, magenta in C, D and grey in C’, D’) antibodies label hemocytes. Only a few hemocytes are attached to the wing discs. Their localizations do not correlate with the positions of the *p35* or *hid-p35* clones.
(TIF)
