# Supplementary figures and images for: Actin remodeling mediates ROS production and JNK activation to drive apoptosis-induced proliferation
Source: PLoS Genet. 2022 Dec 5;18(12):e1010533. doi: 10.1371/journal.pgen.1010533 (PMC9754590; doi:10.1371/journal.pgen.1010533)

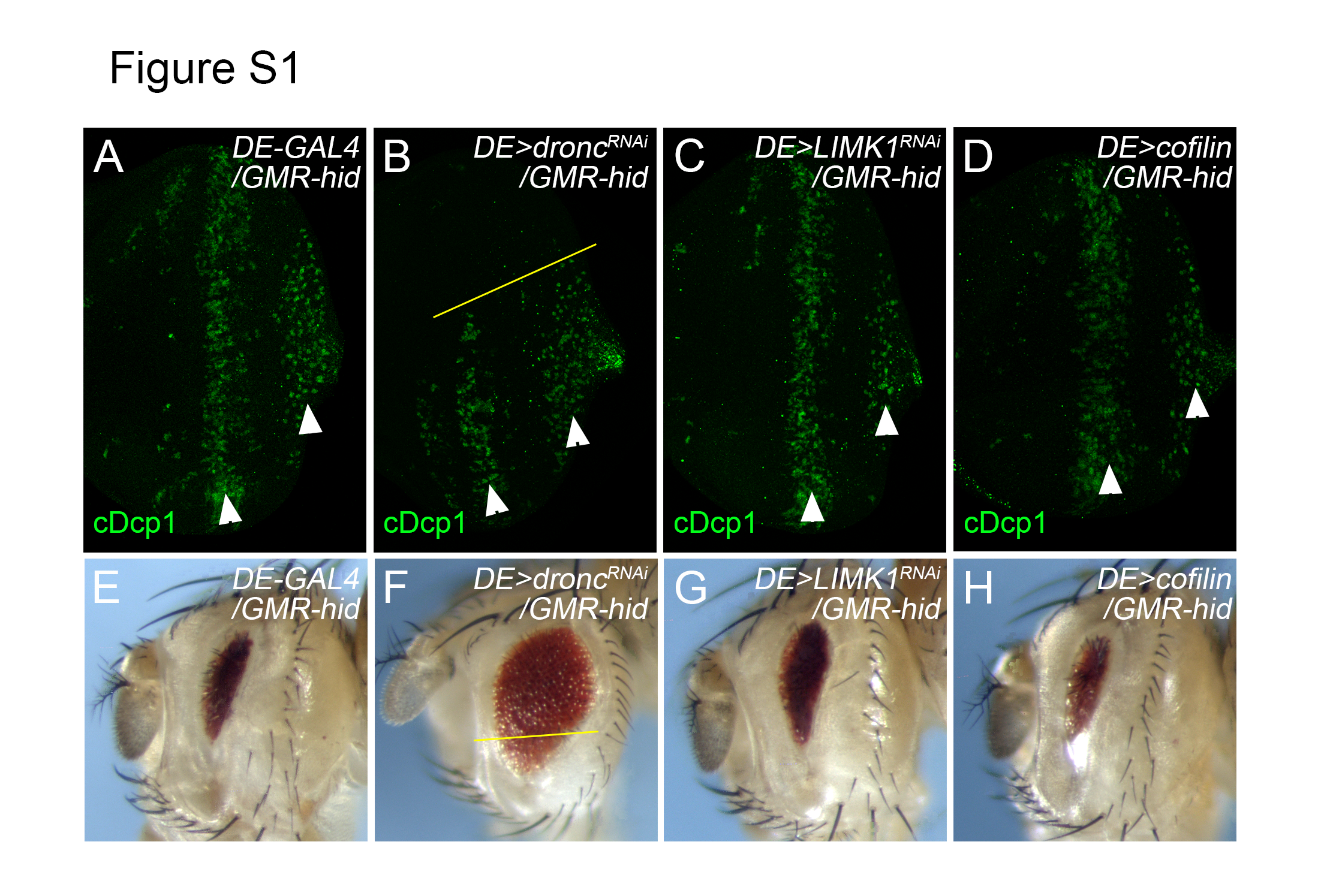

Supplement: S1 Fig — (A-D) Late 3rd instar eye discs expressing a GMR-hid transgene to induce apoptosis, anterior is to the left. DE-GAL4 was used to drive expression of various UAS-constructs in these discs. The cleaved Dcp-1 (cDcp1) antibodies label apoptotic cells. GMR-hid induces two waves of apoptosis as shown in the control (A, arrowheads). Expression of droncRNAi under the control of DE-GAL4 suppresses apoptosis in the dorsal half of the disc (B, highlighted by the yellow dotted line). In contrast, expression of LIMK1RNAi (C) or cofilin (D) does not affect GMR-hid-induced apoptosis. (E-H) Representative adult fly eye images of the indicated genotypes. GMR-hid induces an eye ablation phenotype as shown in the control (E). Expression of droncRNAi driven by DE-GAL4 partially rescues the dorsal part of the eye due to its suppression of GMR-hid-induced apoptosis in this region (F, highlighted by the yellow dotted line). However, expression of LIMK1RNAi (G) or cofilin (H) does not suppress GMR-hid-induced eye ablation phenotype. (TIF) [file pgen.1010533.s001.tif]

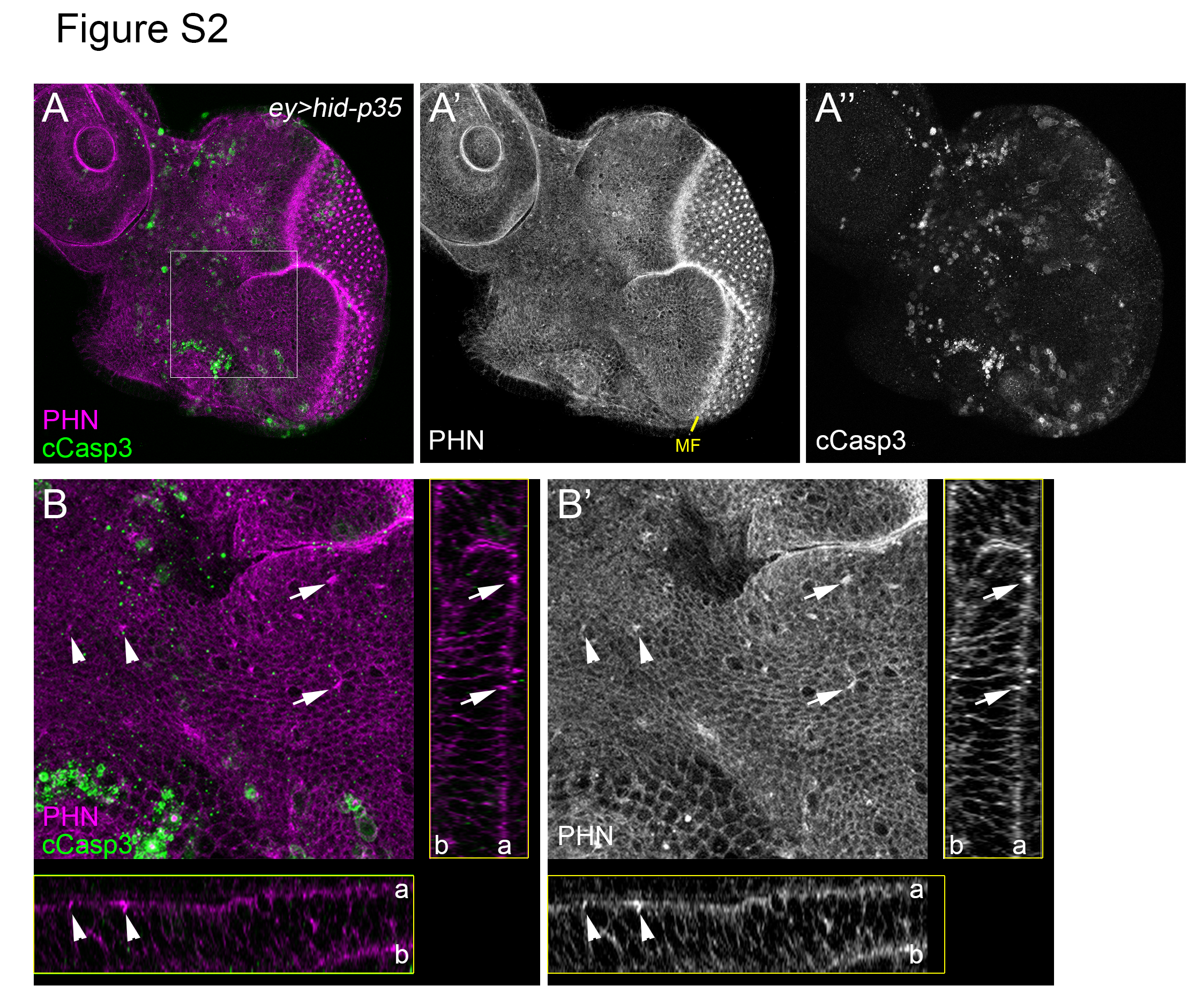

Supplement: S2 Fig — A late 3rd instar ey>hid-p35 disc labelled with PHN (magenta in A, B and grey in A’, B’) and the cleaved caspase-3 antibodies (cCasp3, green in A and grey in A”), a marker of Dronc activity. B and B’ are enlarged images of the outlined area in A, which are shown together with their vertical and horizontal cross sections on right and bottom, respectively. The apical (a) and basal (b) surfaces of these cross sections are as indicated. Four representative F-actin aggregates on these images are indicated by arrows and arrowheads. F-actin accumulation and aggregates are present in the proliferating portion of the disc anterior to the MF (A, A’). Despite a strong activation of Dronc in the same tissue (A, A”), majority of the F-actin aggregates are not overlapping with or adjacent to the cCasp3-positive cells (B). Moreover, these F-actin aggregates localize apically in the disc (arrows and arrowheads in B and B’). (TIF) [file pgen.1010533.s002.tif]

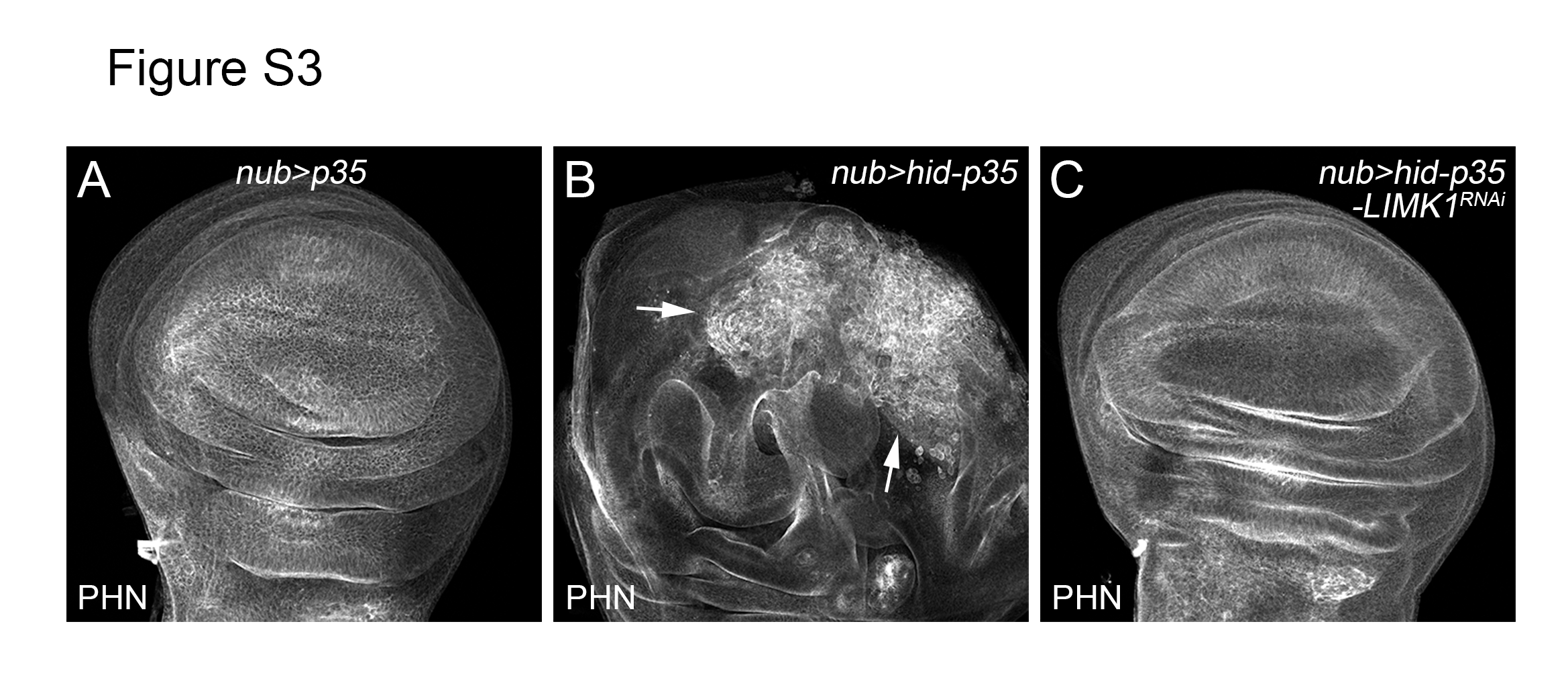

Supplement: S3 Fig — (A-C) Late 3rd instar wing discs labelled with PHN. Anterior is to the left. Compared to the control nub>p35 (A), a massive increase of F-actin filaments was observed in the overgrown wing pouch area of the nub>hid-p35 discs (B, arrows). Such F-actin accumulation and tissue overgrowth were suppressed by a knockdown of LIMK1 with RNAi (C). (TIF) [file pgen.1010533.s003.tif]

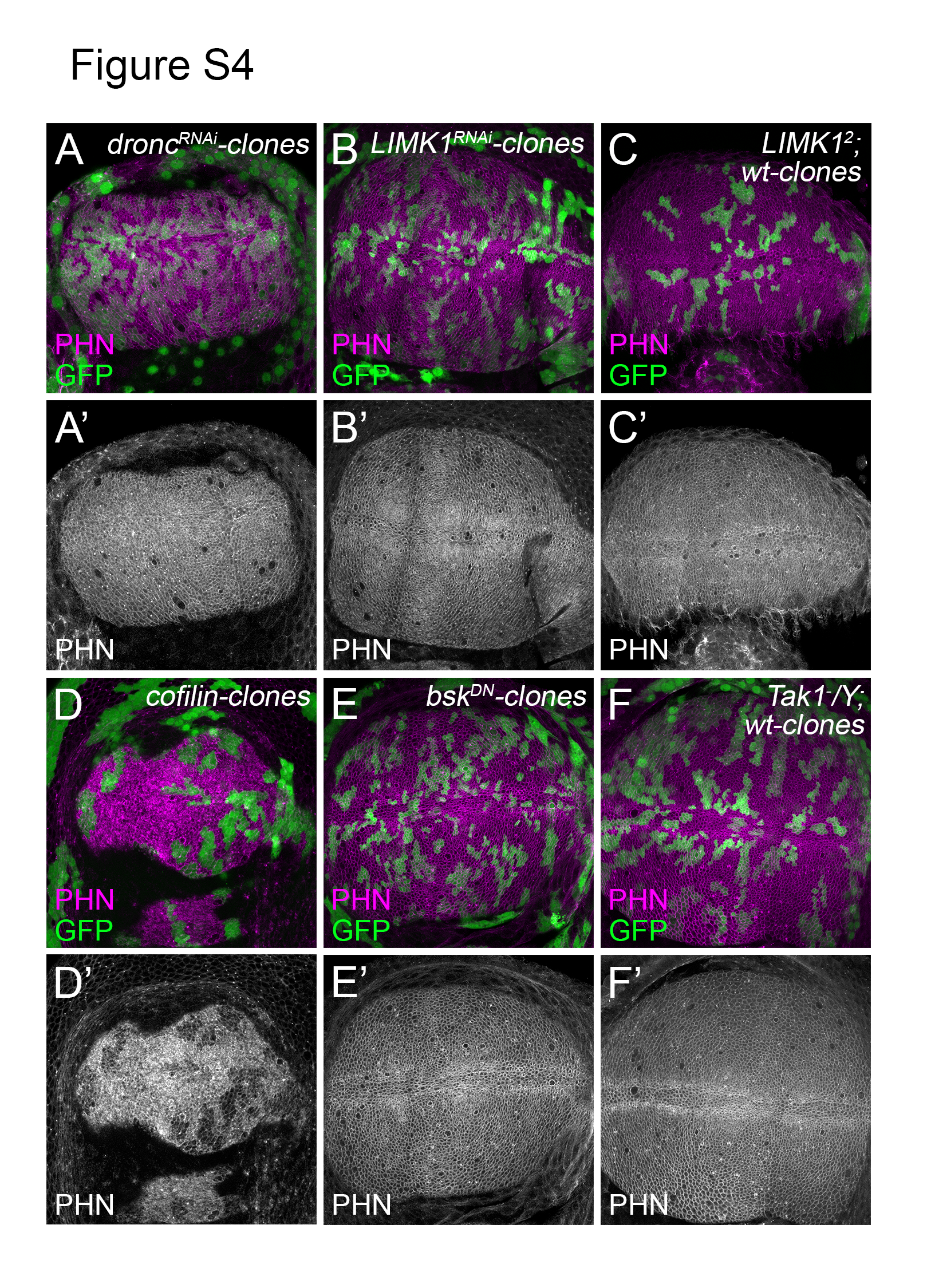

Supplement: S4 Fig — Late 3rd instar wing discs with 48-hour old mosaic clones positively marked by GFP. PHN (magenta in A-F and grey in A’-F’) labels F-actin. Images of the apical part of the discs are shown. The clones expressing droncRNAi (A, A’), LIMK1RNAi (B, B’), or bskDN (E, E’) have no effect on the cortical F-actin pattern in the discs. Similarly, the wildtype clones in the LIMK12 (C, C’) or Tak1 (F, F’) mutant background does not alter the F-actin pattern either. Notably, expression of cofilin (D, D’) reduces the level of F-actin. (TIF) [file pgen.1010533.s004.tif]

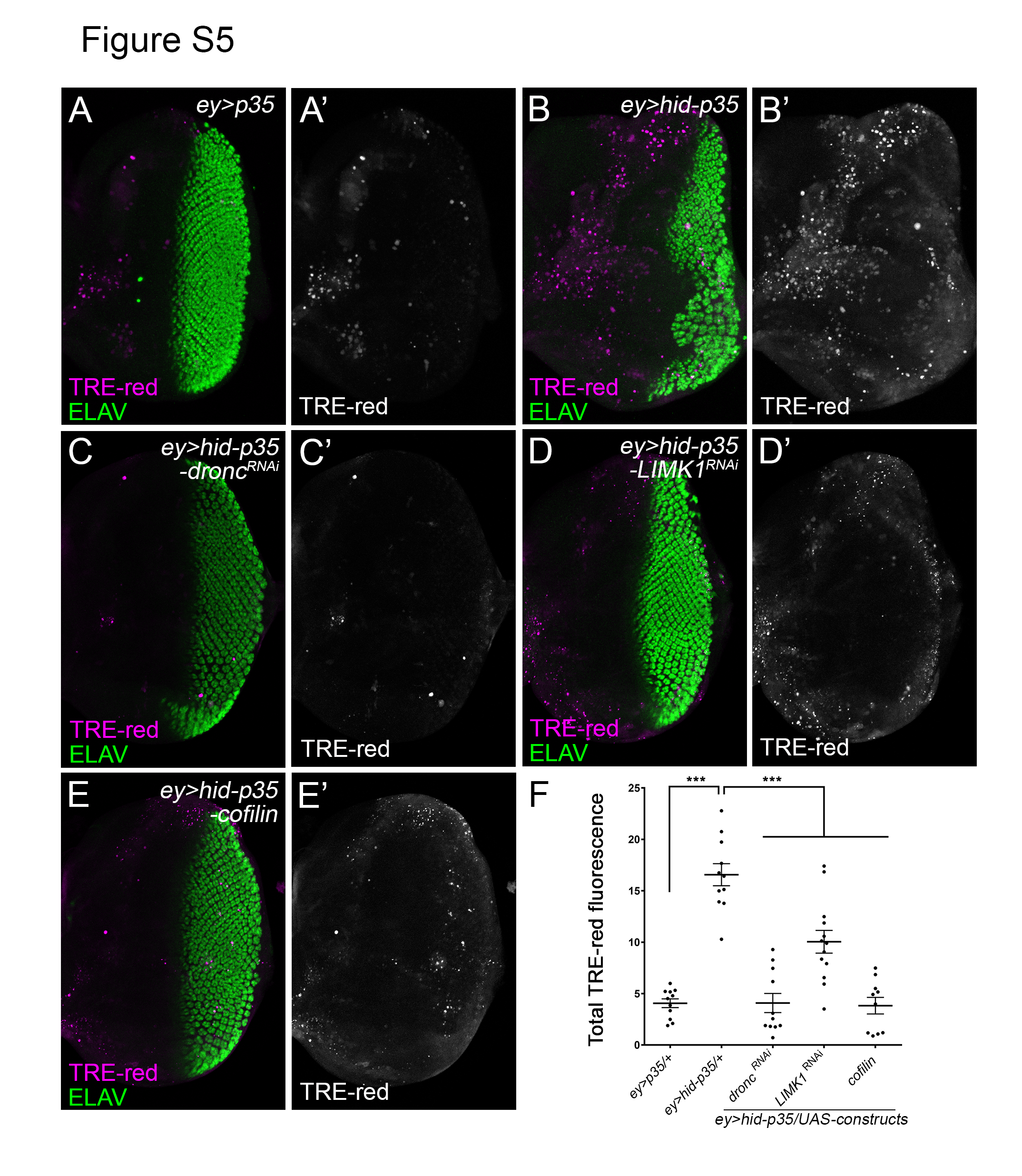

Supplement: S5 Fig — (A-E’) Late 3rd instar eye discs, anterior is to the left. TRE-red (magenta in A-E and grey in A’-E’) is a marker of JNK activity. ELAV (green in A-E) labels photoreceptor neurons therefore indicates the posterior differentiating portion of the eye discs. Compared to the control ey>p35 (A, A’), TRE-red signals strongly increase in the ey>hid-p35 eye discs (B, B’). This increase is suppressed by expressing droncRNAi (C, C’), LIMK1RNAi (D, D’) or cofilin (E, E’). (F) Quantification of the TRE-red signal intensity in the eye discs of the indicated genotypes. Compared to the control ey>p35, the TRE-red signals are significantly (***p < 0.001) increased in the ey>hid-p35 discs. This increase is significantly (***p < 0.001) reduced in response to expression of droncRNAi, LIMK1RNAi or cofilin. (TIF) [file pgen.1010533.s005.tif]

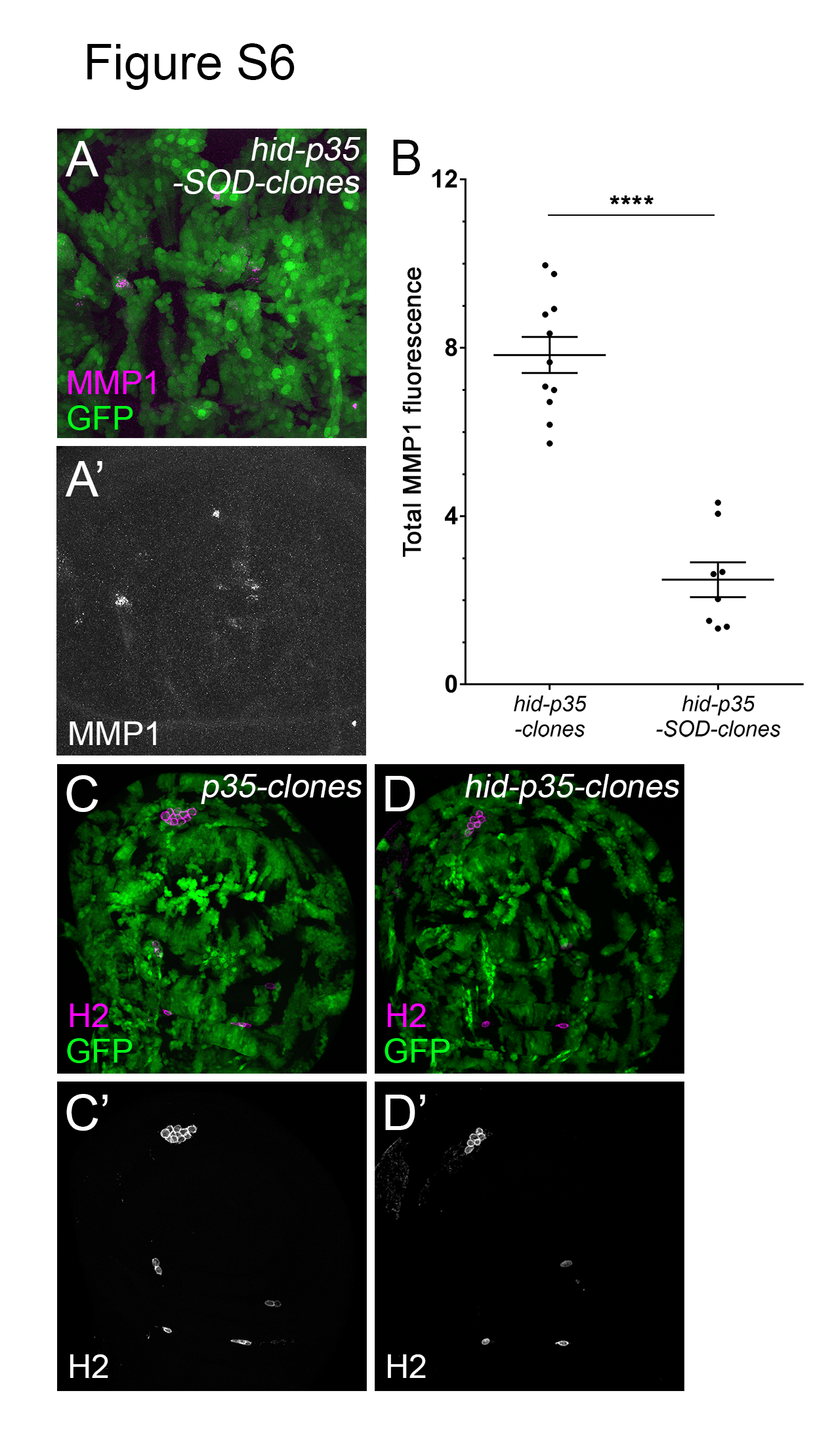

Supplement: S6 Fig — (A-A’) Late 3rd instar wing discs with mosaic clones positively marked by GFP. hid, p35 and SOD are simultaneously expressed in the clones for 48 hours. The JNK activity, indicated by the MMP1 labeling (magenta in A and grey in A’), is very low in these clones. (B) Quantification of the MMP1 signal intensity in the representative wing disc pouches carrying clones of the indicated genotypes. Compared to the discs with hid-p35 clones, the MMP1 signals are significantly (****p < 0.0001) reduced in response to expression of SOD in the clones. (C-D’) Late 3rd instar wing discs with either p35-expressing (C, C’) or hid-p35-expressing (D, D’) clones positively marked by GFP. Anti-Hemese (H2, magenta in C, D and grey in C’, D’) antibodies label hemocytes. Only a few hemocytes are attached to the wing discs. Their localizations do not correlate with the positions of the p35 or hid-p35 clones. (TIF) [file pgen.1010533.s006.tif]
